# Omega-6 and omega-3 oxylipins are implicated in soybean oil-induced obesity in mice

**DOI:** 10.1038/s41598-017-12624-9

**Published:** 2017-10-02

**Authors:** Poonamjot Deol, Johannes Fahrmann, Jun Yang, Jane R. Evans, Antonia Rizo, Dmitry Grapov, Michelle Salemi, Kwanjeera Wanichthanarak, Oliver Fiehn, Brett Phinney, Bruce D. Hammock, Frances M. Sladek

**Affiliations:** 1 Department of Cell Biology and Neuroscience, University of California, Riverside, CA 92521 USA; 2 Davis Genome Center, University of California, Davis, CA 95616 USA; 3 Department of Entomology and Nematology & UCD Comprehensive Cancer Center, University of California, Davis, CA 95616 USA; 4 Creative Data Solutions, Ballwin, MO 63011 USA; 5 National Institutes of Health West Coast Metabolomics Center, University of California, Davis, CA 95616 USA

## Abstract

Soybean oil consumption is increasing worldwide and parallels a rise in obesity. Rich in unsaturated fats, especially linoleic acid, soybean oil is assumed to be healthy, and yet it induces obesity, diabetes, insulin resistance, and fatty liver in mice. Here, we show that the genetically modified soybean oil Plenish, which came on the U.S. market in 2014 and is low in linoleic acid, induces less obesity than conventional soybean oil in C57BL/6 male mice. Proteomic analysis of the liver reveals global differences in hepatic proteins when comparing diets rich in the two soybean oils, coconut oil, and a low-fat diet. Metabolomic analysis of the liver and plasma shows a positive correlation between obesity and hepatic C18 oxylipin metabolites of omega-6 (ω6) and omega-3 (ω3) fatty acids (linoleic and α-linolenic acid, respectively) in the cytochrome P450/soluble epoxide hydrolase pathway. While Plenish induced less insulin resistance than conventional soybean oil, it resulted in hepatomegaly and liver dysfunction as did olive oil, which has a similar fatty acid composition. These results implicate a new class of compounds in diet-induced obesity–C18 epoxide and diol oxylipins.

## Introduction

While humans have been cultivating soybeans for ~5000 years^1^, soybean oil has become a substantial part of our diet only in the last few decades^2^. This increase in soybean oil consumption is due in part to a reaction to large-scale population studies in the 1950s and 60s, which showed that a typical American diet rich in saturated fats from animal products was linked to an increased risk of cardiovascular disease^3,4^. It was subsequently assumed that most if not all saturated fats are unhealthy and conversely that all unsaturated fats are healthy, this despite the ambiguity of evidence of cardio-protective effects of vegetable oils, which are rich in unsaturated fats^5,6^. Similarly, it was assumed that whatever is healthy for the heart is also healthy for the rest of the body although this assumption was never rigorously tested^7,8^. Nonetheless, vegetable oil, and, in particular, soybean oil, began to replace animal fat in the American diet starting in the 1970s, resulting in an exponential rise in soybean oil consumption that parallels the increase in obesity in the U.S. and worldwide^2,9,10^. Indeed, soybean oil is the component in the American diet that has increased the most in the last 100 years^2^. It constitutes >60% of all edible vegetable oil consumption in the U.S^11^. and is ubiquitous in the American diet, especially in cooking oil and processed foods.

Soybean oil is comprised of primarily polyunsaturated fatty acids (PUFAs), particularly linoleic acid (LA, C18:2), an omega-6 (ω6) fatty acid that makes up ~55% of soybean oil. Omega-3 (ω3) fatty acids, especially those found in fish oil, and their ratio to ω6 fatty acids have also received considerable attention. Numerous studies have shown that high ω3:ω6 (and hence low ω6:ω3) ratios are generally healthful^12^. However, like saturated and unsaturated fats, a distinction between different types of ω3 and ω6 fatty acids is often not made, even though this could be relevant to their metabolic effects.

While most experimental diet-induced obesity studies use high fat diets composed of lard or milk fat (rich in saturated fats), a few recent studies (including one from our group) have examined the effects of a diet rich in soybean oil and found that this vegetable oil does in fact increase adiposity, diabetes, insulin resistance and fatty liver^9,13–15^. Furthermore, soybean oil induces more metabolic effects than an isocaloric diet made from coconut oil^13^, which is nearly all saturated fats, albeit of shorter chain length than those in animal fat.

One study proposed, but did not formally prove, that linoleic acid (LA) drives the metabolic effects of soybean, and other vegetable oils^16^. To investigate the role of LA in soybean oil-induced metabolic disease, we compared conventional soybean oil to a new genetically modified (GM) soybean oil (Plenish) which was engineered to generate fewer *trans*-fats by blocking the desaturase gene *FAD2-1* which converts oleic acid (C18:1) to LA^17^ (Supplementary Fig. S1a). The net result is an oil low in LA and high in oleic acid, similar to that of olive oil (Supplementary Fig. S1b), which, as a component of the Mediterranean diet, is considered to be healthful^18,19^. Our results show that the GM oil Plenish does indeed induce less obesity and insulin resistance than conventional soybean oil, although not less diabetes or fatty liver. Plenish also induced hepatomegaly and liver dysfunction, as does olive oil. Importantly, extensive metabolomic and proteomic analyses indicate that oxylipin metabolites of LA and α-linolenic acid (ALA, C18:3ω3) correlate positively with obesity.

## Results

### Genetic modification of soybeans reduces the obesogenic effects of soybean oil

We designed a series of isocaloric, high fat diets with a total fat content similar to that of the American diet (40 kcal%)^20^ (Supplementary Table S1). The control high fat diet is comprised of coconut oil (CO), which is primarily saturated fat and naturally low in LA. The conventional soybean oil diet contains 50% CO and 50% SO (SO + CO) to yield ~10% LA, comparable to that in the current American diet^2,20^ while the PL + CO diet has only 1.4% LA. Normal lab chow (referred to as vivarium chow, Viv) was used as a low fat control and has 1.2% LA. For comparison, the American diet had ~2% LA in the early 1900s^2^.

**Figure 1:**
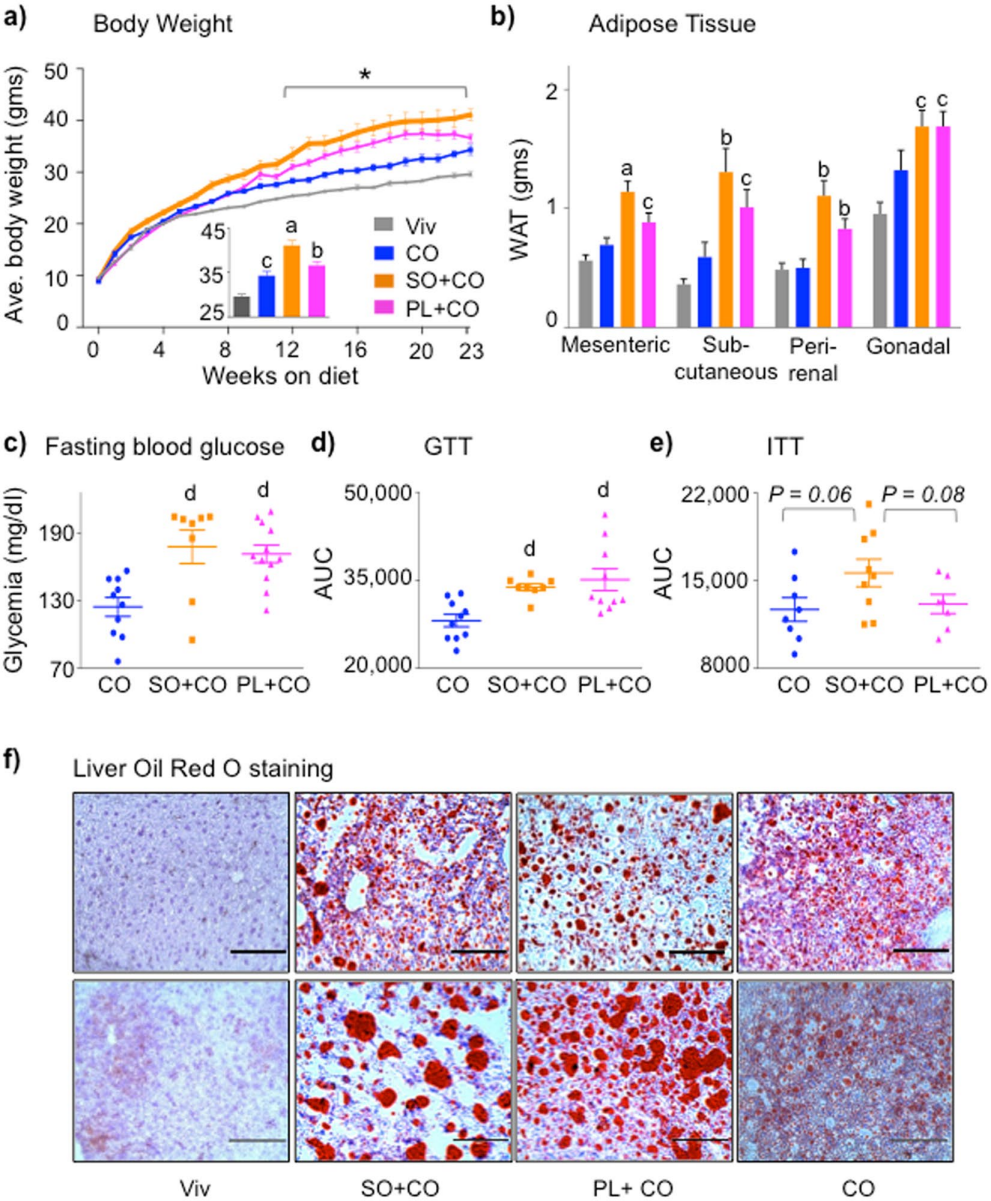
**Plenish induces less obesity and insulin resistance than conventional soybean oil, but similar levels of diabetes and hepatic steatosis.** (**a**) Average weekly body weight of male C57BL/6 N mice on Vivarium chow (Viv) and 40 kcal% high fat diets: CO, coconut oil; SO + CO, conventional soybean oil-enriched; PL + CO, Plenish oil-enriched. Inset, average weight after 23 weeks on diet. N = 12 per group except for Viv (N = 23). *All diets are significantly different from each other, ^a^significantly greater than all others, ^b^than Viv and CO, ^c^than Viv. (**b**) Average weight of white adipose tissues. N = 11–12. ^a^Significantly greater than all others, ^b^than Viv and CO, ^c^than Viv. (**c**) Fasting blood glucose. (**d**) GTT area under the curve (AUC) of mice on diets for 22 weeks. N = 7–12. ^d^Significantly greater than CO. (**e**) ITT AUC of mice on diets for 20 weeks. N = 8-9. (**c**–**e**) See Fig. 6d for Viv values: CO is not significantly different from Viv. ITT AUC: SO + CO vs. Viv (*P* < 0.05). (**f**) Representative Oil Red O staining of livers from mice on the diets for 24 weeks. Scale bar is 100 microns. Each section is from one of 4-6 mice per group. (See Supplementary Fig. S2 for additional stains.) Data are presented as ± SEM (**a**–**e**).

As we observed previously^13^, starting at ~8 weeks on the diet, SO + CO induced significantly greater weight gain than CO in C57BL/6 N male mice, primarily due to increased adipose tissue (Fig. 1a,b) and despite the fact that the two groups of mice had a similar caloric intake (Supplementary Fig. S1c). Importantly, PL + CO caused significantly less weight gain and less adiposity than did SO + CO, although still more than CO (Fig. 1a,b). Both SO + CO and PL + CO induced elevated fasting blood glucose levels and glucose intolerance (Fig. 1c,d and Supplementary Fig. S1d) but only SO + CO increased insulin resistance to near significant levels (*P* = 0.06) (Fig. 1e Supplementary Fig. S1e). An SO only diet also yielded significantly higher insulin resistance (see Fig. 6d).

Since between 30 and 40% of adult Americans have non alcoholic fatty liver disease (NAFLD)^21^, and since fatty liver is a common co-morbidity with obesity, diabetes and insulin resistance, we stained the livers with Oil Red O. PL + CO generated the same striking phenotype of large lipid droplets and hepatocyte ballooning observed previously with SO + CO (Fig. 1f)^13^. In contrast, coconut oil resulted in a less severe fatty liver phenotype (Fig. 1f)^13^: the size and number of lipid droplets were less than in the SO + CO and PL + CO livers.

### Metabolomic analysis reveals a potential role for oxylipins in obesity

**Figure 2:**
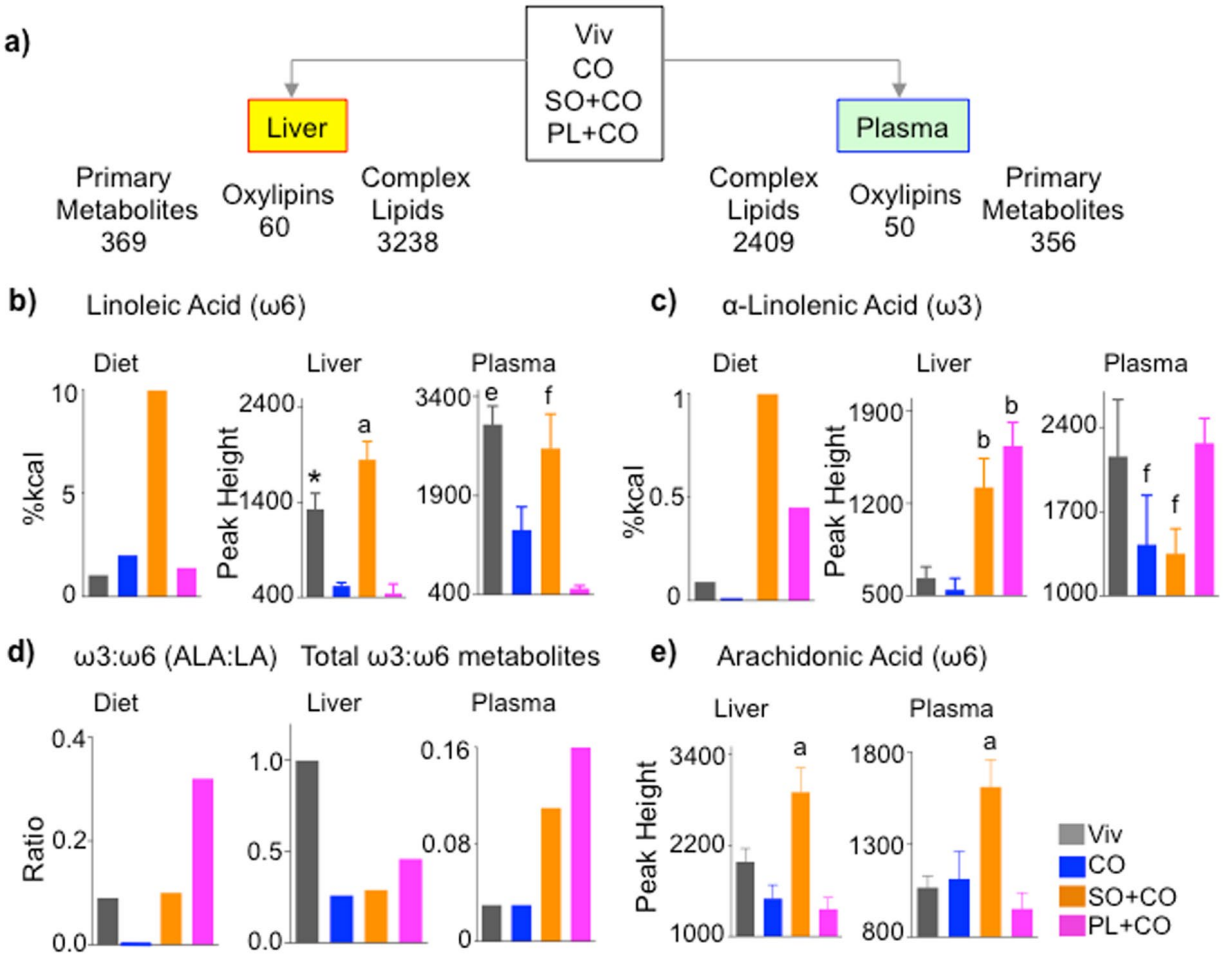
**Metabolomic analysis reveals variations in fatty acid accumulation in liver and plasma between conventional soybean oil and Plenish.** (**a**) Schematic of total number of metabolites identified by the various platforms in liver and plasma of mice fed the indicated diets. **(b,c,e**) Levels of the indicated fatty acids in the diets and liver and plasma of mice fed the respective diets. N = 7–8. (**d**) Ratio of ω3:ω6 fatty acids in diet (ALA:LA) and total ω3:ω6 oxylipins in liver and plasma. *Significantly different from all others. ^a^Significantly different than all others, ^b^than CO and Viv, ^e^than CO and PL + CO, ^f^than PL + CO. Data are presented as ± SEM, except for graphs showing levels or ratios in diets.

To investigate the mechanism by which soybean oil induces its metabolic effects, we performed metabolomics on the liver and plasma of mice fed Viv, CO, SO + CO and PL + CO diets for 24 weeks using three different platforms –primary metabolites, complex lipids and oxylipins (oxidative metabolites of PUFAs^22,23^) (Fig. 2a). We identified 369 primary metabolites in the liver, of which 55 to 75 differed between any two of the high fat diets; 60 oxylipins of which 35 to 49 differed; and 3,238 complex lipids of which ~1,000 to ~1,800 differed. Similar global differences were found in the plasma (Supplementary Fig. S1f and Table S2).

While LA, not surprisingly, was highest in the SO + CO livers, the Viv-fed livers unexpectedly had LA levels that were nearly as high as SO + CO; a similar profile was found in the plasma (Fig. 2b). This could be due to the fact that LA, as an essential fatty acid, is preferentially retained in the body. In contrast, both the CO and PL + CO diets resulted in much lower levels of LA compared to Viv, suggesting that coconut oil may actively impede the accumulation of LA (Fig. 2b).

The other essential fatty acid, ALA, was also highest in the SO + CO diet but its profile differed from that of LA in the liver and plasma. The PL + CO liver accumulated as much ALA as SO + CO livers and the PL + CO plasma had significantly more ALA than SO + CO (Fig. 2c). The PL + CO diet had the highest ω3:ω6 ratio (ALA:LA), a ratio that was maintained in the plasma for total ω3 and ω6 metabolites but reduced in the liver in which Viv chow had the highest ratio (Fig. 2d). Arachidonic acid (AA, C20:4ω6), which is derived from LA and associated with inflammation that often accompanies obesity^24^, was also highest in SO + CO liver and plasma (Fig. 2e). As anticipated, oleic acid was highest in PL + CO liver and plasma and saturated fatty acids abundant in coconut oil–myristic (C14:0) and lauric (C12:0) – were highest in the CO-fed plasma and liver (Supplementary Fig. S3a,c,d). The saturated fat palmitic acid (C16:0) did not vary significantly among any of the diets nor in the plasma, although it was significantly elevated in the SO + CO liver (Supplementary Fig. S3b).

Spearman’s rank correlation coefficient for all annotated complex lipids, primary metabolites, and oxylipins in the livers of mice fed CO, SO + CO or PL + CO revealed 45 primary metabolites (including 14 lipids), 12 complex lipid classes and 16 oxylipins that correlated significantly (*P* < 0.05) with individual values for body weight and total adipose tissue from each mouse (Supplementary Fig. S4a). In contrast, plasma had only half the number of significant correlations compared to the liver (Supplementary Fig. S4b). While the primary metabolites LA and AA correlated positively with body weight and adipose tissue in both liver and plasma, the saturated fats lauric and myristic acid negatively correlated only in the liver (Supplementary Fig. S4a,b). Various complex lipids (e.g., tri- and di-acylglycerides, phosphatidylcholines, and acylcarnitines) correlated either positively or negatively with body/adipose weight in liver and/or plasma. In contrast, oxylipins were the only class of metabolites to show exclusively positive correlations with body and adipose weight in both liver and plasma, with only one exception in the plasma (Supplementary Fig. S4a,b and Table S2).

**Figure 3:**
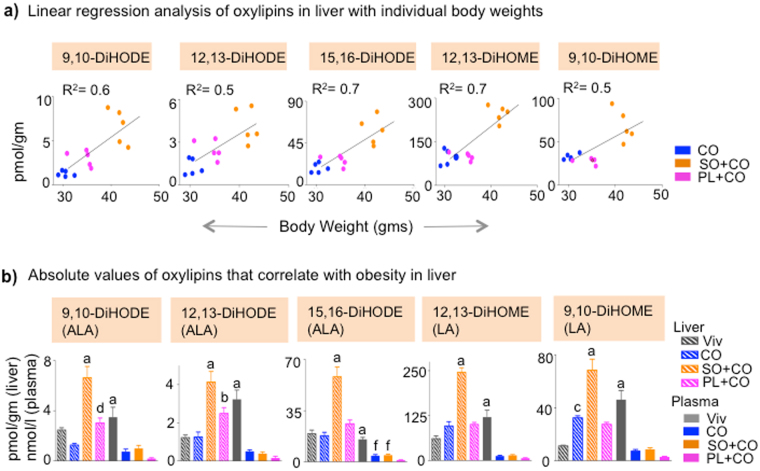
**Liver oxylipins correlate with soybean oil-induced obesity.** (**a**) Correlation between body weight and concentration of liver oxylipins of individual mice. Spearman correlation coefficient (r) is 0.8 for 9,10-DiHODE (*P* = 0.0007), 0.8 for 12,13-DiHODE (*P* = 0.0009), 15,16-DiHODE (*P* = 0.0009), 0.6 for 12,13-DiHOME (*P* = 0.02) and 0.5 for 9,10-DiHOME (*P* = 0.06). Goodness of fit or R^2^ values for linear regression are indicated on the graphs. (The Viv group was not included in the correlation analyses in Supplementary Fig. S4a,b,c). (**b**) Absolute levels of oxylipins that correlate only in liver (hatched bars). Values in plasma (solid bars) are shown as a comparison. N = 4–5 mice per group. ^a^Significantly different (within same tissue) from all others, ^b^from CO and Viv, ^c^from Viv, ^d^from CO, ^f^from PL + CO. The fatty acid from which the oxylipin was derived is shown in parentheses. Data are presented as ± SEM.

Interestingly, nearly all of these oxylipins are generated by the cytochrome P450 (CYP)/soluble epoxide hydrolase (sEH) pathway. A linear regression analysis showed that among all the oxylipins that significantly correlated in CO, SO + CO and PL + CO in Supplementary Fig. S4a,b, there were only four in the liver that had a significant R^2^ (R^2^ ≥ 0.5) (9,10-DiHODE, 12,13-DiHODE, 15,16-DiHODE and 12,13-DiHOME); all four also had a significant Spearman’s coefficient (r ≥ 0.6) (Fig. 3a). A fifth oxylipin (9,10-DiHOME) that missed the *P*-value cut-off in Supplementary Fig. S4a,b also showed a significant R^2^ and Spearman’s coefficient (Fig. 3a) The significance of the linear regression and correlation was maintained or increased when the Viv diet was included (Supplementary Fig. S4c). Interestingly, these five oxylipins are all derived from LA (DiHOMEs) or ALA (DiHODEs) (Supplementary Fig. S5) and were highest in the SO + CO livers (Fig. 3b). Furthermore, their absolute levels were lower in plasma, where they did not correlate significantly with obesity (Fig. 3b).

Another 14 oxylipins had a *P* < 0.05 in the liver or plasma but they did not have a significant R^2^ (Supplementary Fig. S4c–e). They were all higher in liver than plasma and are a mix of metabolites from AA, ALA, eicosapentaenoic acid (EPA, C20:5ω3) and docosahexaenoic acid (DHA, C22:6ω3): EPA and DHA are both derived from ALA. Among these metabolites was 8,9 EpETrE (from AA), the only oxylipin with a negative correlation with body weight (in plasma), consistent with a previous study^25^.

Since the oxylipins of LA and ALA showed a significant, positive correlation with body weight in the liver, we also calculated the linear regression for LA, ALA, and other fatty acids (AA, DHA, oleic acid, palmitic acid, myritstic acid, lauric acid). LA, AA and DHA, which were all elevated in SO + CO livers (Fig. 2b,e and Supplementary Fig. S3e), were the only fatty acids that showed significant R^2^ values (Supplementary Fig. S4f). However, unlike the C18 diols for which the R^2^ values remained (or became more) significant when the Viv chow values were included, the LA, AA and DHA R^2^ values lost their significance (R^2^ ≤ 0.3) when the low-fat diet values were included (Supplementary Fig. S4g).

There were other primary metabolites that were statistically different between SO + CO and PL + CO but none correlated with obesity in the mega analysis (Supplementary Fig. S3e–g, Fig. S4a,b). The level of α-tocopherol, which is enriched in soybean oil, was not significantly different in the Plenish mice (Supplementary Fig. S3h). Taken together, the metabolomic data indicate that CYP/sEH oxylipin metabolites of LA and ALA in the liver (but not the plasma) were the only metabolites to consistently and significantly show a positive correlation with SO-induced obesity.

### Integration of proteomic and metabolomics analysis converges on the CYP/sEH pathway

**Figure 4:**
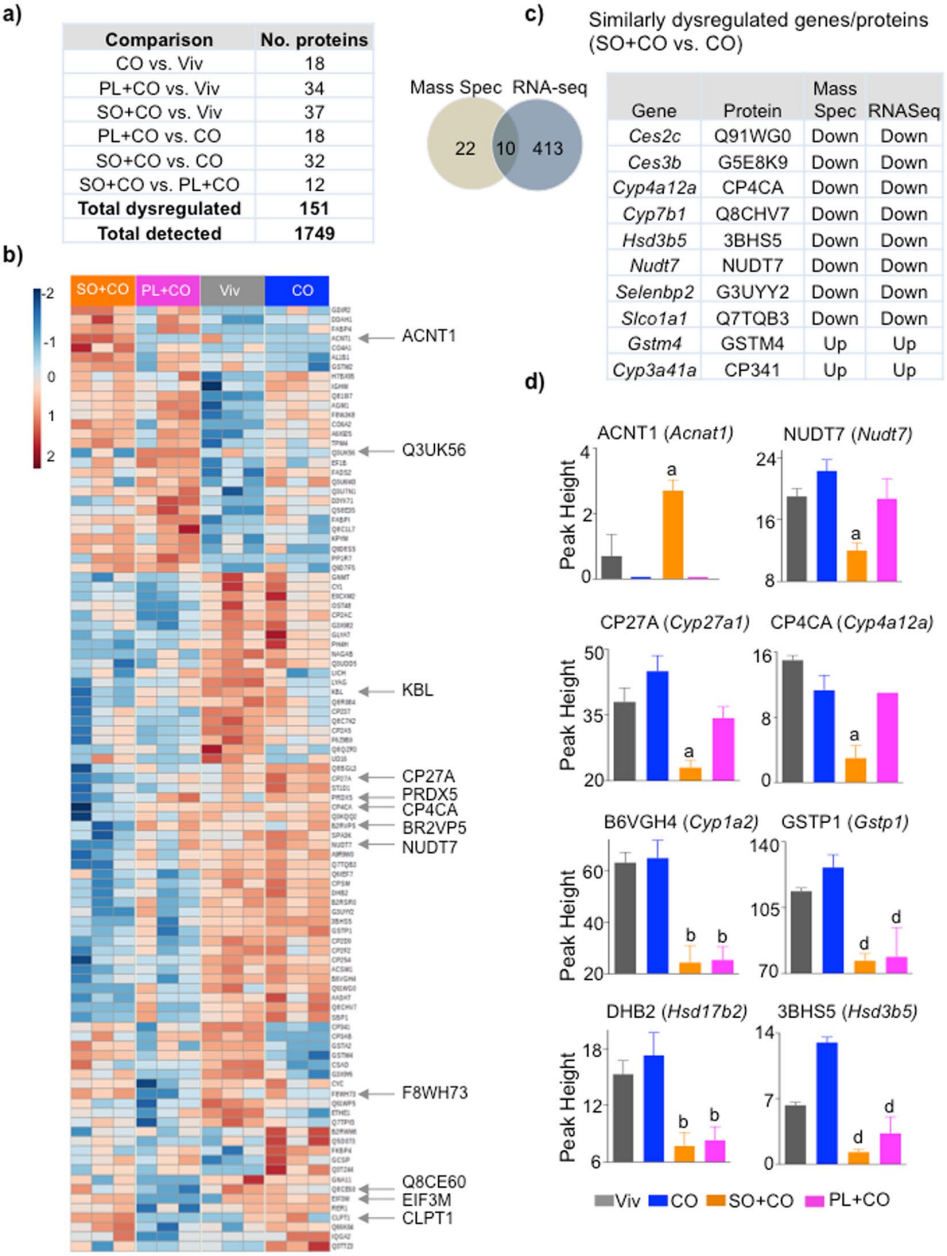
**Proteomic analysis of liver reveals changes induced by both conventional and GM soybean oil.** (**a**) Number of significantly dysregulated proteins in the livers of C57BL/6 N male mice fed the indicated diets (P ≤ 0.05, Tukey’s post hoc test). (**b**) Heatmap showing liver proteins that are significantly different between any two diets. Arrows, significantly different proteins between SO + CO and PL + CO. N = 3 livers per group. (**c**) *Left:* Venn diagram showing overlap of dysregulated proteins between CO and SO + CO fed livers identified by mass spectrometry and RNA-Seq analysis^13^. *Right:* List of similarly dysregulated proteins in SO + CO versus CO livers. (**d**) Proteins that are different in SO + CO or PL + CO compared to any other diet. ^a^Significantly different from all others, ^b^from CO and Viv, ^d^from CO. *P* ≤ 0.05 by One-way ANOVA, Tukey’s post-hoc analysis. Data are presented as ± SEM.

To elucidate the mechanism responsible for the changes in the liver metabolites, we performed proteomics on the livers of mice fed Viv, CO, SO + CO or PL + CO for 24 weeks. Out of 1,749 proteins detected, there were 151 proteins (8.6%) that were significantly dysregulated between any two of the diets (Fig. 4a,b). SO + CO had the greatest number of differences: 37 versus Viv and 32 versus CO as well as 12 proteins that differed between SO + CO and PL + CO, underscoring the effect that dietary oils, especially soybean oil, and even a single modification in a dietary oil (LA to oleic acid), can have on the liver proteome.

Comparison of the proteomic data to our previous RNAseq data from the livers of mice fed SO + CO and CO for 35 weeks^13^ revealed 10 proteins with altered levels in SO + CO versus CO that also had altered mRNA levels. Additional proteins (22 total) that were altered in the proteomic but not the transcriptomic data suggest that non-transcriptional mechanisms may also be implicated (Fig. 4c). Notably, several CYP (1A2, 4A12A, 27 A) and lipid metabolizing enzymes (ACNT1, NUDT7, HSD3B5/17B2) were altered in the SO + CO and PL + CO diets (Fig. 4d). These alterations, as well as that of the Phase II enzyme GSTP1, indicate that different dietary oils lead not only to different fatty acid metabolites, but also to differences in metabolic and detoxification enzymes, which in turn could impact both the metabolomic and xenobiotic profile.

**Figure 5:**
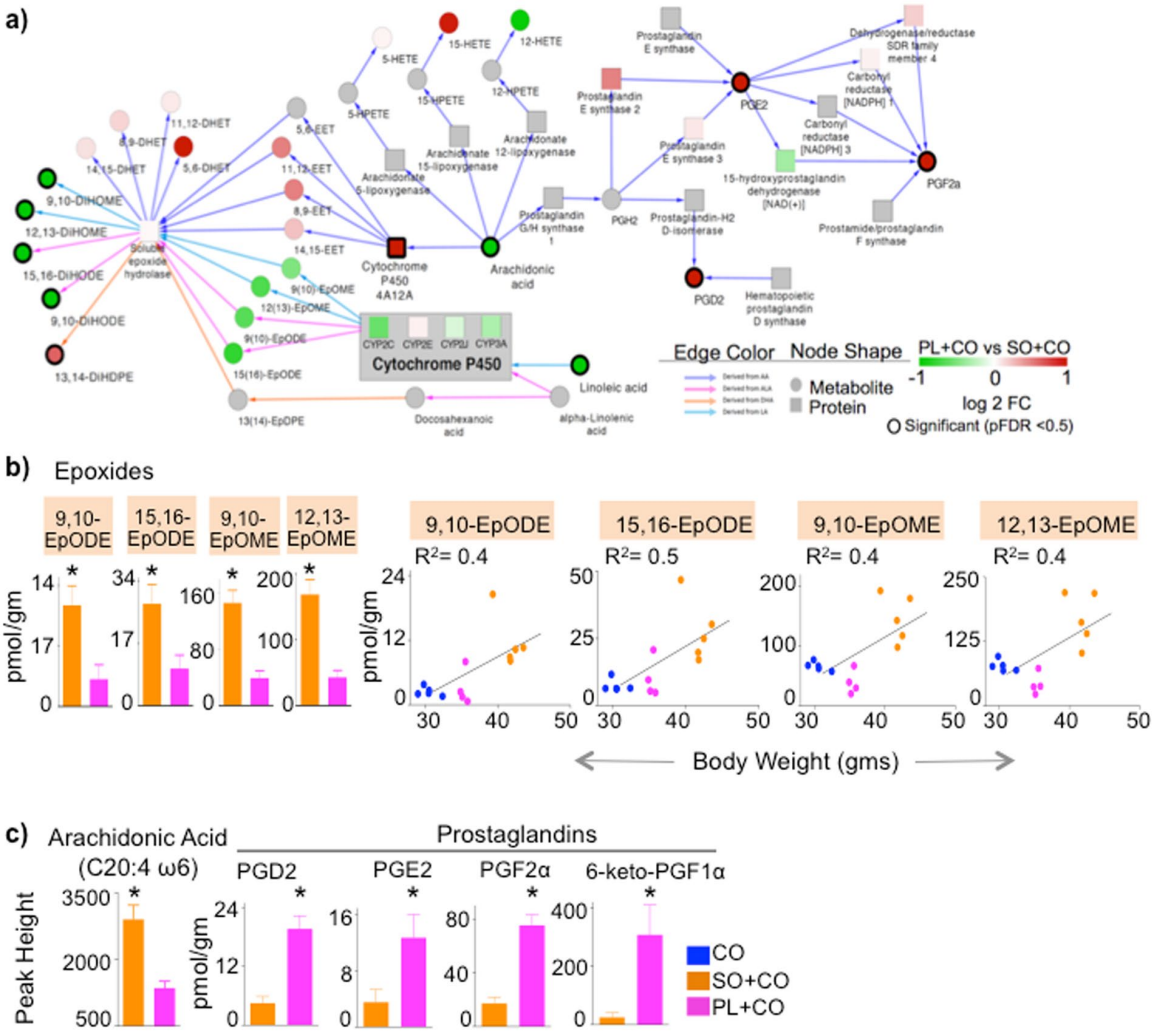
**Alterations in LA-, ALA- and AA-metabolizing enzymes in SO + CO versus PL + CO livers correlate with oxylipin and prostaglandin levels**. (**a**) Integrated proteomic-metabolomic network analysis comparing PL + CO and SO + CO livers based on significant metabolites/proteins (black borders) (pFDR < 0.05) and their connecting nodes. Node colors: red, up in PL + CO; green, up in SO + CO; white, no change; gray, no data mapped to nodes. (**b**) L*eft*, absolute levels of EPOMEs and EPODEs shown in (**a**) (one outlier mouse in the PL + CO group was removed). *Right*, Correlation between body weight and concentration of liver epoxides of individual mice. Spearman correlation coefficient (r) is 0.6 for 9,10-EpODE (*P* = 0.02) and 15,16-EpODE (*P* = 0.03), 0.5 for 12,13-EpOME (*P* = 0.07) and 9,10-EpOME (*P* = 0.08). Goodness of fit or R^2^ values for linear regression are indicated on the graphs. **c)** Levels of arachidonic acid (AA) and prostaglandins in liver. N = 4–5 mice per group. *Significantly different (*P* < 0.05). Data are presented as ± SEM (**b** and **c**).

A network analysis generated by cross-referencing the liver SO + CO versus PL + CO proteome with their respective metabolomes revealed a modest, albeit insignificant, down regulation in Plenish of CYP2C and CYP3A families, which metabolize LA and ALA to EpOME and EpODE epoxides, respectively (Fig. 5a). Although the change in individual CYP enzymes did not reach significance, the combined effect was sufficient to decrease the C18 epoxide levels in PL + CO livers; this decrease reached significance once an outlier was removed (Fig. 5b left). Importantly, linear regression analysis showed a modest positive correlation of the C18 epoxides with body weight (Fig. 5b right, R^2^ > 0.4). The epoxides in turn are converted by sEH to C18 diols (Supplementary Fig. S5a,c), which strongly correlated with body weight (Fig. 3) and were significant in the network analysis (Fig. 5a). Although the C18 epoxides and diols were impacted by the diets, the sEH activity was largely unaffected as shown by the similar diol:epoxide ratios between PL + CO and SO + CO. Only the 9,10-DiHODE/EpODE ratio was statistically different in the liver (Supplementary Fig. S5b).

CYP4A12A was significantly up regulated in PL + CO versus SO + CO livers (Figs 4d and 5a). It hydroxylates AA to epoxyeicosatrienoic acids (EpETrEs or EETs), which in turn are converted by EPHX2 to dihydroxytrienoic acids (DiHETrEs or DHETs)^26^. Both EETs and DHETs were elevated in PL + CO relative to SO + CO (Fig. 5a), albeit not significantly, and have been reported to have anti-obesogenic properties^25^.

Finally, several prostaglandins (PGD2, PGE2, PGF2α and 6-keto-PGF1α) were significantly elevated in PL + CO livers, whereas AA, from which they are derived, was higher in SO + CO livers (Fig. 5a,c). The increases could be explained by the modest (but not significant) increase in prostaglandin E synthase 2 (*Ptges2)* and dehydrogenase/reductase SDR family member 4 (*Dhrs4*) (Fig. 5a), demonstrating again that small changes in enzyme levels can have significant effects on metabolites.

Taken together, these results implicate both ω3 and ω6 hepatic C18 oxylipins (epoxides and diols) derived from the essential fatty acids ALA and LA in obesity induced by conventional soybean oil. In contrast, AA-derived prostaglandins do not correlate positively with obesity, but were elevated in Plenish livers.

### Plenish induces similar effects to olive oil, including hepatomegaly and liver dysfunction

**Figure 6:**
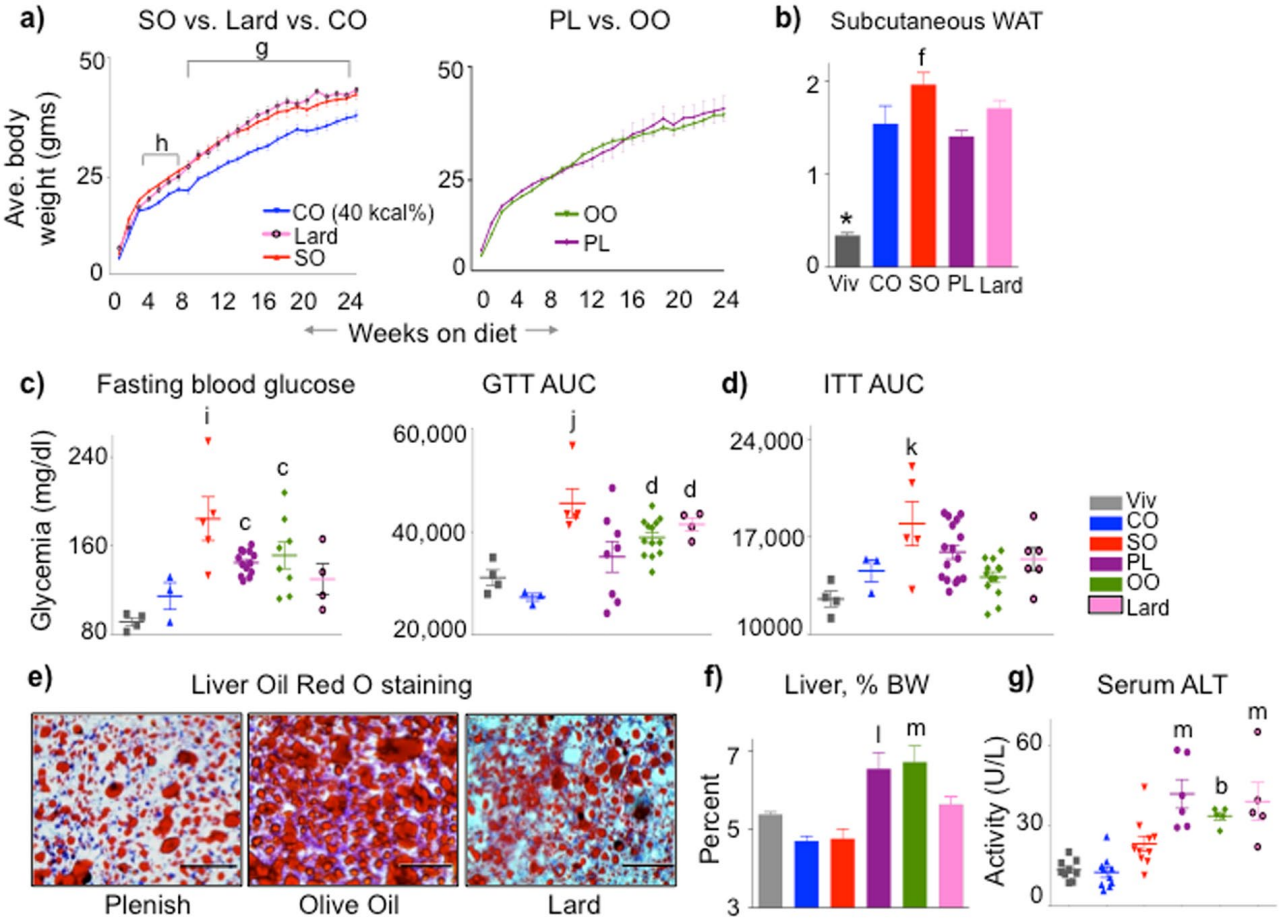
**Plenish induces similar metabolic effects as olive oil; conventional soybean oil is similar to lard.** (**a**) Average weekly body weights of C57BL/6 N male mice started on the indicated diets at weaning. High fat diets (35 kcal%) with a single fat source: SO, soybean oil only; PL, Plenish oil only; OO, olive oil only. CO (40 kcal%) is as in Fig. 1. N = 7–16. ^g^CO significantly different from SO and lard, or ^h^from SO. (**b**) Average mass of subcutaneous (flank) fat pads from mice on diets for 24 weeks. *Significantly different from all others, ^f^from PL. (**c**) Fasting blood glucose (18–20 weeks). N = 10–13. ^i^Significantly greater than Viv, CO and lard, ^c^than Viv. AUC, area under the curve of a GTT assay (18-20 weeks). N = 4–13. ^j^Significantly greater than CO, PL and Viv or ^d^than CO. (**d**) AUC of ITT (18 weeks). N = 5-12 except CO (N = 3) and Viv (N = 4). ^k^Significantly greater than Viv and OO. (**e**) Representative Oil Red O staining of livers. Scale bar is 100 microns. N = 4–6 per group. (See Supplementary Fig. S6e for SO and Supplementary Fig. S2 for additional sections) (**f**) Liver weight as percent body weight. N = 10–13. (See Supplementary Fig. S6g for absolute liver body weight) (**g**) Serum ALT activity. N = 5–10. For (**f**) and (**g**), ^l^significantly greater than all others except OO, ^m^than SO, CO and Viv or ^b^than Viv and CO. All data are presented as ± SEM.

To rule out potential confounding effects of the coconut oil in the diets, we reformulated the diets to include only a single source of fat (just soybean oil or Plenish) and compared them to isocaloric diets made with olive oil or animal fat (lard) (35% kcal total fat) (Supplementary Table S1). The conventional soybean oil-only diet (SO) induced an identical weight gain and adiposity to lard while the Plenish-only diet (PL) was identical to olive oil (OO), despite comparable food intake (Fig. 6a,b and Supplementary Fig. S6a,b,c). The SO, PL and OO diets, but not lard or CO, all produced elevated fasting glucose levels with SO inducing the highest level (Fig. 6c left). SO, OO and lard induced glucose intolerance, with SO again having the largest effect (Fig. 6c right and Supplementary Fig. S6d). Interestingly, the conventional soybean oil diet was still the only one to induce insulin resistance (Fig. 6d and Supplementary Fig. S6e).

There were unanticipated effects on liver morphology and function: while all four diets induced fatty livers with large lipid droplets and hepatocyte ballooning (Fig. 6e, Supplementary Figs S2 and S6f), mice fed PL or OO but not CO, SO or lard, had excessive liver weights (Fig. 6f and Supplementary Fig. S6g). These mice, along with those on the lard diet, also had significantly reduced liver function, as determined by elevated levels of circulating alanine transaminase (ALT) (Fig. 6g). Taken together, these results indicate that the fatty liver phenotype does not always track with obesity, diabetes or insulin resistance. They also show that the genetic modification of soybean oil may induce detrimental health effects in terms of liver function even though it induces less obesity and insulin resistance than conventional soybean oil.

## Discussion

This is the first report to compare the metabolic effects of conventional soybean oil to those of GM oil (Plenish) with low LA but high oleic acid. It is also the first study to compare the metabolomic and proteomic profiles induced by these oils high in unsaturated fats to those generated by an oil rich in saturated fatty acids (coconut oil). Out of >3,000 known compounds, the only class of metabolites that consistently correlated positively with obesity across all three high fat diets (CO, SO + CO, and PL + CO) were the oxylipins of both ω-6 LA and ω-3 ALA generated by the CYP/sEH pathway. The correlation was primarily in the liver, not the plasma, which could have clinical implications.

**Figure 7:**
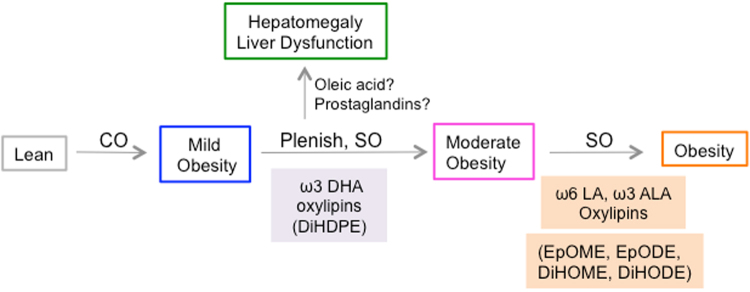
**Proposed model for the role of hepatic oxylipin metabolites in diet-induced obesity**. CO, coconut oil; PL, Plenish (high oleic acid), low linoleic acid (LA); SO, conventional soybean oil (high LA). Prostaglandins, PGD2, PGE2, PGF2α, 6-keto-PGF1α. Oxylipin boxes are color-coded with other figures (see Supplementary Fig. S5c for overview); box outlines of phenotypes are color-coded with diets (Figs 1 and 6). See text for details.

Based on these results, we propose a model for diet-induced obesity that is divided into three steps or stages that are modulated by the availability of different types of fatty acids and their metabolites (Fig. 7). In the first stage, coconut oil (CO) high in medium chain saturated fats induces mild obesity. This could be due simply to the greater number of calories in the CO diet compared to the Viv chow, since the saturated fats in CO did not correlate with body weight (Supplementary Fig. S3b–d and Fig. S4f). Importantly, mice on the high fat diet consisting of coconut oil alone do not progress beyond this first stage of metabolic disease even after long-term feeding (up to 35 weeks): they do not develop diabetes or insulin resistance, only moderately fatty liver^13^.

In the second stage, mice fed the high soybean oil diets (either conventional or Plenish) developed more obesity. DiHDPE metabolites of ω3 DHA generated by the CYP/sEH pathway, which are significantly elevated in both SO + CO and PL + CO livers, may play a role in this second phase (Fig. 7, Supplementary Fig. S4c,d). However, they did not correlate with obesity across all the diets and if anything tended to be higher in Plenish than conventional soybean oil, raising the possibility that the DiHDPEs could also have a positive effect. Indeed, certain DiHDPEs are referred to as resolvins for their anti-inflammatory effects^27^.

The third stage correlates with different CYP/sEH metabolites. Oxylipins of ω3 ALA (9,10-, and 15,16-EpODE; 9,10-, 12,13- and 15,16-DiHODE) and ω6 LA (9,10- and 12,13-EpOME; 9,10- and 12,13-DiHOME) were all significantly increased in the livers of mice fed conventional soybean oil compared to Plenish and correlated positively with body weight across all three high fat diets (Figs 3, 5b and 7). Finally, Plenish and olive oil, both rich in oleic acid, caused liver dysfunction and hepatomegaly (Figs 6f,g and 7). We could find no relevant literature about olive oil affecting liver size or ALT levels and studies on olive oil and hepatic steatosis do not reach a consensus^28^. It is also possible that the elevated prostaglandin levels in the PL + CO liver play a role since prostaglandins have been shown to regulate hepatic growth either directly^29^ or indirectly via their interaction with peroxisome proliferator-activated receptors (PPARs)^30^ Fig. 5c and 7).

Another category of oxylipins generated by the 12/15 LOX pathway–metabolites of AA (LXA4 and 9-HETE) and ALA (9-HOTrE)–may also play a role, although they did not reach statistical significance between conventional and Plenish soybean oil, only between SO + CO and CO or Viv chow (Supplementary Fig. S4c). LXA4 and 9-HETE are both elevated in the plasma of humans with metabolic syndrome: 9-HETE was suggested as a causal factor in oxidative stress, while LXA4 is thought to be involved in the down regulation of inflammation^31^. Although we could find no reports of 9-HoTRE in metabolic syndrome, 13-HoTRE has been cited as an anti-inflammatory oxylipin^32^. The only non-enzymatic oxylipin detected was EKODE (12,13-epoxy-9-keto-10(trans)-octadecenoic acid), which was slightly higher in SO + CO versus PL + CO in the liver but not significantly different from CO (Supplementary Table S2).

Oxylipins in general, as bioactive signaling lipids, are increasingly being associated with inflammation, vascular permeability, and cardiovascular disease as well as diabetes, obesity-induced hypertriglyceridemia, and insulin signaling^23,33–36^. However, we found only two published reports on C18 diols and obesity. One report found a negative correlation between obesity and esterified LA/ALA-derived oxylipins^37^ while the other observed a positive correlation with non esterified (free) oxylipins^38^, which are the ones we analyzed since they are considered to be bioactive^23,39^. Many of the negative effects of epoxy derivatives of LA, such as cytotoxicity and inflammation, are actually attributed to their sEH metabolites such as 12,13-DiHOME^40–45^. EpODEs were also recently linked to the obese phenotype in humans^31^. In contrast, not much is known about the biological action of the ALA-derived DiHODEs although it has been reported that 9,10-DiHODE and 12,13-DiHODE concentrations are lower in serum of hyperlipidemic men compared to normolipidemic men^46^. Additional investigation of the role of the C18 oxylipins in obesity and other aspects of the metabolic syndrome – diabetes, insulin resistance, hepatocyte ballooning and large lipid droplets–is clearly warranted.

Our results also show that, just as not all saturated and unsaturated fats have the same effects, not all ω3 fatty acids may be healthful, since we found a positive correlation between ω3 (ALA) oxylipins and obesity. Furthermore, even though the proper balance of ω6:ω3 fatty acids in the diet is often emphasized^12,47^, we found that Plenish and olive oil have identical metabolic effects even though they have very different ω6:ω3 ratios (3.4 and 10.0, respectively) (Supplementary Table S1). Interestingly, all the oxylipins that correlated well with obesity are derived from fatty acids that must be obtained from the diet (LA and ALA), suggesting that a dietary overload of even essential fatty acids can have significant implications for health.

The vast majority of diet-induced obesity studies use lard as the source of fat and assume that they are looking at the effects of saturated fat, as well as cholesterol. In the U.S., lard comes from animals that are typically fed soybean meal^48^ and consequently the levels of LA in lard can be quite high (11% or higher)^12,49^. Hence, it is possible that some of the metabolic effects in the literature attributed to saturated fats in these lard-based studies could actually be due to high LA from soybean oil, as others have found with farmed salmon^14^. It will be of interest to determine whether the C18 oxylipins identified in this study are also elevated in the livers of animals fed conventional high fat diets based on animal fat. In terms of cholesterol, contrary to the widely held belief that PUFAs such as those in soybean oil lower plasma cholesterol levels, in our experiments neither the conventional nor the GM soybean oil ameliorated the increase in plasma cholesterol induced by coconut oil. (The increase in cholesterol with coconut oil has been reported previously^50–52^). Additionally, both soybean oils increased cholesteryl esters in the liver (Supplementary Fig. S3i,j), consistent with a number of recent reports debunking the putative cholesterol lowering effects of vegetable oils^8,53^.

In summary, we show that while the GM soybean oil Plenish induces less obesity and insulin resistance than conventional soybean oil in mice, it also produces negative effects on liver function, as does olive oil. Our results also implicate various ω6 as well as ω3 oxylipin metabolites of ALA and LA in obesity, although it remains to be determined if they act in a causal fashion.

## Methods

### Diets

Three isocaloric diets with 40 kcal% fat and four isocaloric diets with 35%kcal fat were formulated in conjunction with Research Diets, Inc. (New Brunswick, NJ) (Supplementary Table S1). Normal lab chow referred to as Vivarium diet (Viv) was included as a low-fat control. See Supplemental Experimental Procedures for more details on diet formulation.

### Animals

Male C57BL/6 N mice (Charles River Laboratories) were weaned at three weeks of age and assigned randomly to one of the diets. The animals were maintained on a 12:12 h light-dark cycle in a specific pathogen free vivarium (SPF) for the 40 kcal% diet feeding experiment. The mice in the 35 kcal% experiment (Fig. 6 and Supplementary Fig. S6) and the mice used for the 24-week vivarium (Viv) chow liver oxylipin analysis were housed in a non-SPF facility. At least 12 mice were put on each diet with three to four animals per cage. Food intake was recorded twice a week on a per cage basis; individual mouse weights were recorded once a week (Supplementary Figs S1c and S6c).

### Ethics Statement

Care and treatment of animals was in accord with guidelines from and approval by the University of California Riverside Institutional Animal Care and Use Committee (AUP#20140014). All mice had *ad libitum* access to food and water (other than the indicated fasting times). At the end of the study, mice were euthanized by carbon dioxide inhalation (before noon), in accordance with stated NIH guidelines.

### Glucose and Insulin Tolerance Tests

Glucose tolerance (GTT) and insulin tolerance tests (ITT) were performed as described previously^13^.

### Alanine Transaminase (ALT) Activity Test

Blood for the Alanine Transaminase (ALT) colorimetric assay to measure liver disease or injury^54^ was collected by cardiac puncture (without anti-coagulant) and allowed to clot at room temperature for 30 min, followed by centrifugation at 9,300 × g for 15 min at 4 °C. Serum was stored immediately at −80 °C. The assay and data analysis were done according to manufacturer’s instructions (Catalog#: 700260 Cayman Chemicals, Ann Arbor USA).

### Tissue Samples and Staining

Liver was collected, stored and analyzed by Oil Red O staining as described previously^13^.

### Metabolomic, Lipidomic, Oxylipin and Proteomic Analysis

For analysis of primary metabolites, 30 µL of plasma or 5 mg of liver tissue homogenate were extracted and derivatized; metabolite levels were quantified by chromatography time-of-flight (GC-TOF) mass spectrometry as previously described^55^. The precipitated protein from the primary metabolite analysis was used for the proteomic analysis. For analysis of complex lipids, plasma aliquots (20 µL) or liver tissue homogenates (5 mg) were extracted using a modified liquid-liquid phase extraction approach proposed by Matyash *et al*.^56^. For analysis of non-esterified oxylipins, plasma aliquots (250 µL) or liver tissue homogenates (100 mg) were extracted and analyzed according to previously described protocols^56,57^. Epoxyeicosatrienoic acids are referred to as EpETrEs or EETs and dihydroxytrienoic acids are referred to as DiHETrEs or DHETs. See Supplementary Information for details on the sample collection and metabolomic and proteomic analysis and Supplementary Table S2 for all the datasets, including separate datasheets for primary metabolites, complex lipids, oxylipins and proteomics results for the plasma and liver as well as information sheets for each platform. Raw metabolomics and proteomics data is available on Metabolomics Workbench and Massive/Proteome Exchange, respectively. See Supplementary Information for accession numbers.

### Network-based analysis

An integrated network of metabolites and proteins was computed by Grinn software tool^58^, an R-based tool that integrates biochemical and genomic relationships from several databases, including KEGG^59^, Reactome^60^ and ENSEMBL^61^. We used significant metabolites and proteins (pFDR < 0.05 comparing PL + CO vs SO + CO for liver at 24 weeks) to infer the metabolite-protein networks. The resulting networks were visualized in Cytoscape^62^.

### Statistical Analysis

Data are presented as mean +/− standard error of mean (SEM). Statistical significance, using GraphPad Prism version 6 for Mac, is defined as *P* ≤ 0.05 using the following tests: Two-way ANOVA with Holm-Sidak post hoc analysis for differences in weight gain over time among the different diets. One-way ANOVA with Holm-Sidak post hoc analysis was performed for tissue weights at harvest and GTT, ITT and ALT assays.

For metabolomics data, values were log2 transformed and statistical significance was determined using a One-Way ANOVA. Specific group differences were determined using Tukey HSD post hoc test. ANOVA *P*-values were adjusted using Benjamini and Hochberg false-discovery rate adjustment. Statistical analyses were conducted using R statistical software. For major structural lipids, the summed intensities of all lipids belonging to that specific lipid class (e.g., triacylglycerides) were used. Lipids were delineated by degree of saturation. Saturated: <2 or <4 double bonds present in lipid species that contain one or more acyl chains, respectively. Unsaturated: ≥2 or ≥4 double bonds present in lipid species that contain one or more acyl chains, respectively. For correlations between metabolites and metabolic phenotypes, Spearman’s rank correlations on log10-transformed values of known compounds were performed; only significant correlations are included (*P* ≤ 0.05) (Supplementary Fig. S4). Linear regression analysis was performed between body weight and concentration of oxylipins or fatty acids in the liver. The following cut-offs were used to determine significance: Spearman’s coefficient r > 0.5 with *P* ≤ 0.05 and R^2^ > 0.5 (Figs 3, 5b and Supplementary Fig. S4c–f). For proteomics data, One-way ANOVA values were log10-transformed and statistical significance was determined using One-way ANOVA. Hierarchical clustering was calculated on Euclidean Distance with Ward’s agglomeration (Fig. 4). For the integrated network analysis, significance was based on one-way ANOVA on log10-transformed data. Benjamini and Hochberg tests were used for FDR adjustment. Tukey’s HSD was used to determine specific group differences (Fig. 5).

## Electronic supplementary material

Supplemenatary Information

Dataset 1
