# Supplementary material for: Omega-6 and omega-3 oxylipins are implicated in soybean oil-induced obesity in mice
Source: Sci Rep. 2017 Oct 2;7:12488. doi: 10.1038/s41598-017-12624-9 (PMC5624939; doi:10.1038/s41598-017-12624-9)
Supplement: Supplementary file 1 — Supplemenatary Information [file 41598_2017_12624_MOESM1_ESM.pdf]

Supplementary Information

Omega-6 and omega-3 oxylipins are implicated in soybean oil-induced obesity in mice

Poonamjot Deol[a]*, Johannes Fahrmann[b]*, Jun Yang[c], Jane R. Evans[a], Antonia Rizo[a], Dmitry Grapov[d], Michelle Salemi[b], Kwanjeera Wanichthanarak[e], Oliver Fiehn[e], Brett Phinney[b], Bruce D. Hammock[c], Frances M. Sladek[a]

*Co-first authors

[a] Department of Cell Biology and Neuroscience, University of California, Riverside, CA 92521 USA
[b] Davis Genome Center, University of California, Davis, CA 95616 USA
[c] Department of Entomology and Nematology & UCD Comprehensive Cancer Center, University of California, Davis, CA 95616 USA
[d] Creative Data Solutions, Ballwin, MO 63011 USA
[e] National Institutes of Health West Coast Metabolomics Center, University of California, Davis, CA 95616 USA

Corresponding author:
Dr. Frances M. Sladek
2115 Biological Sciences Building, University of California, Riverside, CA 92521
E-mail: frances.sladek@ucr.edu
Tel: (951)-827-2264.

Short title:

PUFA metabolites correlate with soybean oil-induced obesity

Supplementary Experimental Procedures

Diets

Three isocaloric diets with 40 kcal% fat (4.87 kcal/gm) (Supplementary Table S1) were formulated in conjunction with Research Diets, Inc. (New Brunswick, NJ). The diets are based on the Surwit diet, which is widely used in diet-induced obesity studies and formulated with elements from the AIN-93 diet. The 5% fiber from cellulose in the AIN diet is replaced with cornstarch [1]. The coconut oil diet (CO) is identical to the basic high fat diet ("HFD") in our previous study (see Deol et al., 2015 in main text references). These 40 kcal% diets were: CO (36 kcal% from coconut oil and 4 kcal% from soybean oil to provide the essential fatty acids LA and ALA); SO+CO (21 kcal% fat calories from coconut oil and 19 kcal% from soybean oil, of which 10 kcal% were from LA ("LA-HFD" in (Deol et al., 2015)); and PL+CO (conventional soybean oil was replaced on a per gram basis with the genetically modified (GM) High Oleic Soybean Oil Plenish (DuPont Pioneer, Johnston, IA)).

The fatty acid compositions of Plenish, conventional soybean oil and coconut oil used in the diets were determined by Covance Laboratories (Madison, WI) (Supplementary Table S1) (values for lard and olive oil are from Research Diets). The LA content of Plenish was confirmed to be 7.42% versus 52.9% for SO (similar to values reported in the literature). The level of ALA was also reduced in Plenish resulting in a ω6:ω3 ratio of 3.4 in Plenish versus a ratio of 8.1 in conventional soybean oil (Supplementary Table S1). In contrast, the ω6:ω3 ratio in olive oil was 10 although the absolute levels of the fatty acids were reduced to 6% for LA and 0.6% for ALA. The phytosterol composition of Plenish and conventional soybean oil was determined by Covance Laboratories and found to be essentially identical (data not shown).

We formulated four additional diets with 35 kcal% total from a single source: soybean oil (SO), Plenish (PL), olive oil (OO) or lard (Supplementary Table S1). (Total fat content was reduced from 40 kcal% to ensure that the diets would pellet.) Like coconut oil, lard is high in saturated fatty acids although its fatty acid composition is different from that of coconut oil (Supplementary Table S1). The total amount of carbohydrates and protein were constant across all the diets, including the low fat control: vivarium (Viv) chow (Purina Test Diet 5001, Newco Distributors, Rancho Cucamonga, CA) with 3.36 kcal/gm fat. Viv chow also contains ~25% fiber.

Diets were provided in pellet form, twice weekly for up to 24 weeks; the amount of food consumed was monitored weekly on a per cage basis. Mice consumed similar amounts of CO, SO+CO and PL+CO (both in terms of mass and kcal), indicating that differences in total calorie intake were not responsible for the differences in weight gain (Supplementary Fig. S1c). Mice also consumed similar amounts of SO, PL and lard diets (Supplementary Fig. S7b). Only the OO-fed mice had a somewhat higher caloric intake than PL-fed mice. Diets were provided in pellet form, twice weekly for up to 24 weeks; the amount of food consumed was monitored weekly on a per cage basis.

Sample Collection for metabolomics and proteomic analysis

Liver tissue from the large lobe was snap frozen and stored in liquid nitrogen. Cardiac blood was collected using syringes (BD 3ml Luer-Lok Tip, #309657) and needles (Precision Glide needles, 26Gx5/8, 305115) rinsed with 0.5M EDTA in 1.5-ml Eppendorf tubes and kept on ice for 30 min, followed by centrifugation at 9300xg for 5 min at 4°C. Three aliquots were made from the plasma: two were stored immediately at -80°C for primary metabolite and complex lipid analysis, while the third for

oxylipin analysis was treated with a 1:50 dilution of an antioxidant solution (0.2% triphenylphosphine (TPP), 0.2% butylated hydroxytoluene (BHT) and 1% EDTA) before freezing at -80°C. The MiniX database [2] was used as a Laboratory Information Management System (LIMS) and for sample randomization prior to all analytical procedures. All samples were analyzed in one batch.

Primary Metabolite Analysis

Liver tissue was homogenized using a GenoGrinder 2010. The plasma or liver homogenates were extracted using ice-cold 'degassed' 3:3:2 (v/v/v) acetonitrile, isopropanol and ultrapure water. Internal standards, C8–C30 fatty acid methyl esters (FAMEs), were added to samples and derivatized with methoxyamine hydrochloride in pyridine and subsequently by MSTFA (Sigma-Aldrich) for trimethylsilylation of acidic protons and analyzed by GC-TOF mass spectrometry. An Agilent 7890A gas chromatograph (Santa Clara, CA) was used with a 30 m long, 0.25 mm i.d. Rtx5Sil-MS column with 0.25 µm 5% diphenyl film; an additional 10 m integrated guard column was used (Restek, Bellefonte PA) [3-5]. A Gerstel MPS2 automatic liner exchange system (ALEX) was used to eliminate sample cross-contamination during the GC-TOF analysis. Samples (0.5 µL) were injected at 50°C (ramped to 250°C) in splitless mode with a 25 sec splitless time. The chromatographic gradient consisted of a constant flow of 1 ml/min, ramping the oven temperature from 50°C for to 330°C over 22 min. Mass spectrometry was done using a Leco Pegasus IV time of flight mass (TOF) spectrometer, 280°C transfer line temperature, electron ionization at −70 V and an ion source temperature of 250°C. Mass spectra were acquired at 1525 V detector voltage at *m/z* 85–500 with 17 spectra/sec.

All samples were analyzed in one batch, throughout which data quality and instrument performance was monitored using quality control and reference plasma samples (National Institute of Standards and Technology, NIST). Quality controls, comprised of a mixture of standards and analyzed every 10 samples, were monitored for changes in the ratio of analyte peak heights, and used to ensure equivalent instrumental conditions ($P<0.05$, *t*-Test comparing observed to expected ratios of analyte response factors) over the duration of the sample acquisition [6]. Acquired spectra were further processed using the BinBase database [2,7].Briefly, output results [3] were filtered based on multiple parameters to exclude noisy or inconsistent peaks. Detailed criteria for peak reporting including mass spectral matching, spectral purity, signal-to-noise and retention time are discussed in detail elsewhere [8]. Known artifact peaks such as polysiloxanes or phthalates were excluded from data export in BinBase. Missing values were replaced by investigating the extracted ion traces of the raw data, subtracted by the local background noise. All entries in BinBase were matched against the Fiehn mass spectral library of 1,200 authentic metabolite spectra using retention index and mass spectrum information or the NIST11 commercial library. Metabolites were reported if present in at least 50% of the samples for any given dietary group. Data reported as quantitative ion peak heights were normalized by the sum intensity of all annotated metabolites and used for further statistical analysis.

Complex Lipid Analysis

Briefly, 225 µl of chilled methanol containing an internal standard mixture (PE(17:0/17:0); PG(17:0/17:0); PC(17:0/0:0); C17 Sphingosine; C17 Ceramide; SM (d18:0/17:0); Palmitic Acid-d3; PC (12:0/13:0); Cholesterol-d7; TG (17:0/17:1/17:0)-d5; DG (12:0/12:0/0:0); DG (18:1/2:0/0:0); MG (17:0/0:0/0:0); PE (17:1/0:0); LPC (17:0); LPE (17:1)) and 750 µL of chilled MTBE (Methyl Tertiary Butyl Ether, Sigma Aldrich) containing the internal standard 22:1 cholesteryl ester was added to 20 µL or 5 mg aliquots of sample. Samples were shaken for 6 min at 4°C using an Orbital Mixing Chilling/Heating Plate (Torrey Pines Scientific Instruments), thereafter 188 µL of ultrapure water were added. Samples were vortexed, centrifuged and the upper layer was transferred to a new 1.5-mL

Eppendorf tube. The upper layer was dried under reduced pressure, resuspended in methanol:toluene (90:10) containing 50 ng/mL CUDA ((12- [[(cyclohexylamino)carbonyl]amino]- dodecanoic acid, Cayman Chemical), sonicated, centrifuged and subsequently transferred to an amber glass vial (National Scientific-C4000-2W) with a micro-insert (Supelco 27400-U).

Resuspended samples were analyzed on an Agilent 1290A Infinity Ultra High Performance Liquid Chromatography system with an Agilent Accurate Mass-6530-QTOF in both positive and negative mode. The column (65°C) was a Waters Acquity UPLC CSH C18 (100mm length x 2.1mm internal diameter; 1.7 µM particles) coupled with a Waters Acquity VanGuard CSH C18 1.7 µM Pre-column. For positive mode acquisition, the solvent system included A) 60:40 v/v acetonitrile:water (LCMS grade) containing 10 mM ammonium formate and 0.1% formic acid and B) 90:10 v/v isopropanol:acetonitrile containing 10 mM ammonium formate and 0.1% formic acid. For negative mode acquisition, the solvent system consisted of A) 60:40 v/v acetonitrile:water (LCMS grade) containing 10 mM ammonium acetate and B) 90:10 v/v isopropanol:acetonitrile containing 10 mM ammonium acetate. The gradient started from 0 min 15% (B), 0-2 min 30% (B), 2-2.5 min 48% (B), 2.5-11 min 82% (B), 11-11.5 min 99% (B), 11.5-12 min 99% (B), 12-12.1 min 15% (B), and 12.1-15 min 15% (B). The flow rate was 0.6 mL/min and with an injection volume of 5 µL for ESI (+/-) mode acquisitions. ESI capillary voltage was +3.5 kV and -3.5 kV with collision energies of 25eV and 40eV for MSMS collection in positive and negative acquisition modes, respectively. Data were collected at a mass range of m/z 60-1700 Da with a spectral acquisition speed of 2 spectra per sec. Data quality and instrument performance were monitored throughout the data acquisition using quality control (internal STDS), method blanks and reference pooled plasma samples.

Data were processed using MZmine 2.10. All peak intensities are representative of peak heights. Annotations were completed by matching experimental accurate mass MS/MS spectra to MS/MS libraries, including Metlin-MSMS, NIST12 and LipidBlast [9]. Spectral matching was automated using the MSPepSearch tool, and manually curated using The NIST Mass Spectral Search Program Version 2.0g. Metabolite libraries were created, in positive and negative ionization modes, containing all confirmed identified compounds. MZmine's Custom Database Search tool was used to assign annotations based on accurate mass and retention time matching. Data, reported as peak heights for the quantification ion (m/z) at the specific retention time for each annotated and unknown metabolite, was normalized to the class-specific internal standard (annotated) or to the internal standard which had the closest retention time (unknowns) or to tissue weight extracted (liver homogenates). Pooled Bioreclamation plasma (BioreclamationIVT) and method blanks were used to assess data quality.

Oxylipin Analysis

Briefly, samples were extracted by solid phase extraction and analyzed by ultrahigh performance liquid chromatography tandem mass spectrometry (UPLC-MS/MS) (Agilent 1200SL-AB Sciex 4000 QTrap). Analyst software v.1.4.2 was used to quantify peaks according to corresponding standard curves with their corresponding internal standards. Oxylipin concentrations are presented as nmols/liter in plasma and pmol/gm in tissue.

See Supplementary Table S2 for complete primary metabolites, complex lipids and oxylipin datasets.

Proteomic Analysis

The protein pellet from the samples used for primary metabolite analysis was solubilized in 200 µL of 6 M urea. Dithiothreitol (DTT, 200 mM) was added to a final concentration of 5 mM and samples were incubated for 30 min at 37°C. Next, 20 mM iodoacetamide (IAA) was added to a final

concentration of 15 mM and incubated for 30 min at room temperature, followed by the addition of 20 µL DTT to quench the IAA. Trypsin/Lys-C (Promega) was added to the sample and incubated for 4 hours at 37°C. Samples were then diluted to >1M urea by the addition of 50 mM AMBIC digested overnight at 37°C. The following day, samples were desalted using Macro Spin Column (Nest Group).

Digested peptides were analyzed by LC-MS/MS on a Thermo Scientific Q Exactive Orbitrap Mass spectrometer in conjunction Proxeon Easy-nLC II HPLC (Thermo Scientific) and Proxeon nanospray source. The sample run order was block randomized to reduce sampling bias. The digested peptides were loaded on a 100 micron x 25 mm Magic C18 100Å 5U reverse phase trap where they were desalted online before being separated using a 75 micron x 150 mm Magic C18 200Å 3U reverse phase column. Peptides were eluted using a 180-min gradient with a flow rate of 300 nl/min. An MS survey scan was obtained for the m/z range 300-1600; MS/MS spectra were acquired using a top 15 method, where the top 15 ions in the MS spectra were subjected to HCD (High Energy Collisional Dissociation). An isolation mass window of 2.0 m/z was for the precursor ion selection, and normalized collision energy of 27% was used for fragmentation. A 5-sec duration was used for the dynamic exclusion.

Tandem mass spectra were extracted and charge state deconvoluted by Proteome Discoverer (Thermo Scientific). All MS/MS samples were analyzed using X! Tandem (The GPM, thegpm.org; version TORNADO (2013.02.01.1)). X! Tandem was set up to search Uniprot Mouse database (September 2014, 16,976 Proteins), the cRAP database of common laboratory contaminants (www.thegpm.org/crap; 114 entries) plus an equal number of reverse protein sequences assuming the digestion enzyme trypsin. X! Tandem was searched with a fragment ion mass tolerance of 20 PPM and a parent ion tolerance of 20 PPM. Iodoacetamide derivative of cysteine was specified in X! Tandem as a fixed modification. Deamidation of asparagine and glutamine, oxidation of methionine and tryptophan, sulphone of methionine, tryptophan oxidation to formylkynurenin of tryptophan and acetylation of the n-terminus were specified in X! Tandem as variable modifications.

Criteria for protein identification

Scaffold (version Scaffold_ 4.0.6.1, Proteome Software Inc., Portland, OR) was used to validate MS/MS based peptide and protein identifications. Peptide identifications were accepted if they exceeded specific database search engine thresholds. X! Tandem identifications required at least –Log (Expect Scores) scores of greater than 1.2 with a mass accuracy of 5 ppm. Protein identifications were accepted if they contained at least 2 identified peptides. Using the parameters above, the Decoy False Discovery Rate (FDR) was calculated to be 1.1% on the protein level and 0.0% on the spectrum level [10]. Proteins that contained similar peptides and could not be differentiated based on MS/MS analysis alone were grouped to satisfy the principles of parsimony.

The proteomic results were based on total spectral counts. Count data for ~7370 proteins were evaluated for missing or zero values and only those proteomic measurements whose spectral count median was > 0 were included (2416 out of 7367 [33%] were kept). This was followed by annotation to known genes -- only those proteomic measurements with a mapped gene ID were kept (nothing was removed). Finally, replicate precision was done in which redundant proteins were removed; those with highest median precision were kept (1749 out of 2416 total).

Counts were log10 normalized and statistical significance determined with One-way ANOVA with Benjamini and Hochberg FDR adjustment. Tukey HSD was used to determine specific group differences. See Supplementary Table S2 for complete proteomics dataset.

Supplementary References

1 Reeves, P. G., Nielsen, F. H. & Fahey, G. C., Jr. AIN-93 purified diets for laboratory rodents: final report of the American Institute of Nutrition ad hoc writing committee on the reformulation of the AIN-76A rodent diet. *J Nutr* **123**, 1939-1951 (1993).

2 Scholz, M. & Fiehn, O. SetupX--a public study design database for metabolomic projects. *Pacific Symposium on Biocomputing. Pacific Symposium on Biocomputing*, 169-180 (2007).

3 Kind, T., Tolstikov, V., Fiehn, O. & Weiss, R. H. A comprehensive urinary metabolomic approach for identifying kidney cancerr. *Analytical Biochemistry* **363**, 185-195 (2007).

4 Fiehn, O. Extending the breadth of metabolite profiling by gas chromatography coupled to mass spectrometry. *Trends in Analytical Chemistry* **27**, 261-269 (2008).

5 Weckwerth, W., Wenzel, K. & Fiehn, O. Process for the integrated extraction, identification and quantification of metabolites, proteins and RNA to reveal their co-regulation in biochemical networks. *Proteomics* **4**, 78-83 (2004).

6 Fiehn, O. *et al.* Quality control for plant metabolomics: reporting MSI-compliant studies. *The Plant Journal : for Cell and Molecular Biology* **53**, 691-704 (2008).

7 Fiehn, O., Wohlgemuth, G. & Scholz, M. Setup and annotation of metabolomic experiments by integrating biological and mass spectrometric metadata. *Lect Notes Comput Sc*, 224-239 (2005).

8 Fiehn, O., Wohlgemuth, G. & Scholz, M. in *Data Integration in the Life Sciences* Vol. 3615 (ed Raschid L Ludäscher B) 224-239 (Springer, 2005).

9 Kind, T. *et al.* LipidBlast in silico tandem mass spectrometry database for lipid identification. *Nature Methods* **10**, 755-758 (2013).

10 Tabb, D. L. What's driving false discovery rates? *Journal of Proteome Research* **7**, 45-46 (2008).

Supplementary Figure S1

a)

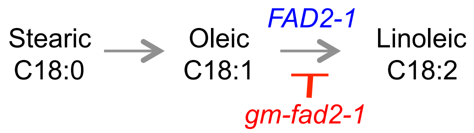

b)

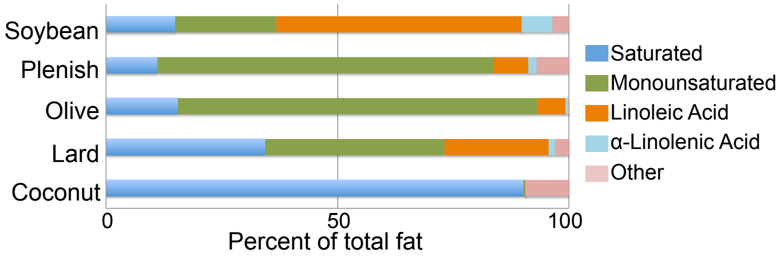

c)

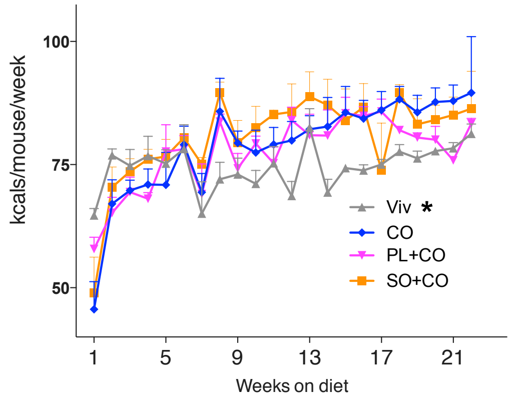

d)

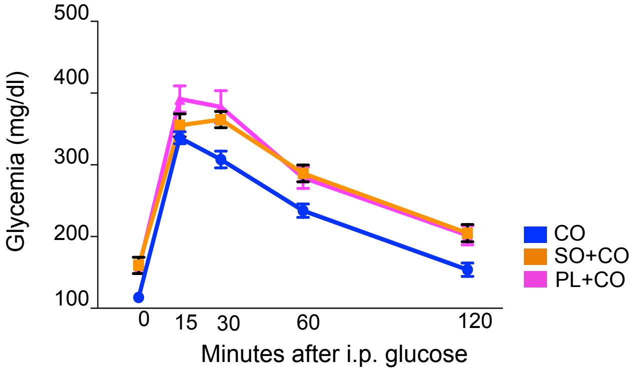

e)

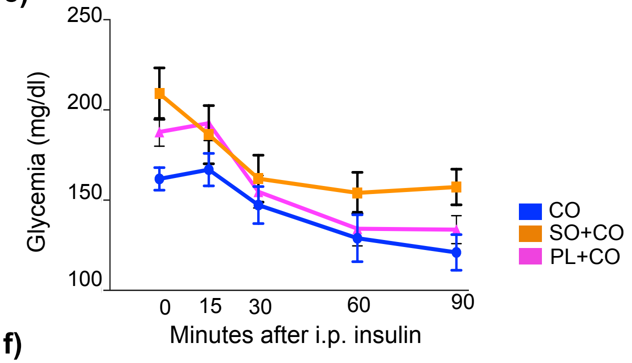

f)

Plasma	CO vs. Viv	SO+CO vs. CO	PL+CO vs. CO	SO+CO vs. PL+CO
Primary Metabolites	121	62	45	47
Complex Lipids	1745	748	1195	1389
Oxylipins	42	24	36	40
**Total**	**1908**	**834**	**1276**	**1476**

Liver	CO vs. Viv	SO+CO vs. CO	PL+CO vs. CO	SO+CO vs. PL+CO
Primary Metabolites	59	75	55	62
Complex Lipids	1916	1854	988	1797
Oxylipins	55	46	49	35
**Total**	**2030**	**1975**	**1092**	**1894**

Supplementary Figure S1. Supplemental information for mice on 40 kcal% fat diets.

a) Genetic modification in High-Oleic Soybean Oil (Plenish). **b)** Approximate fatty acid composition of oils used in this study. See Table S1 for detailed amounts and percentages. **c)** Average weekly food consumption of C57BL/6N male mice on 40 kcal% fat diets on a per cage basis. N= 8-12 mice (3 to 4 cages) per diet. * Mice on Viv diet consumed significantly fewer kcals over the course of the experiment compared to mice on the CO and SO+CO diets (One-way ANOVA, Holm-Sidak post hoc analysis). **d)** GTT of mice on diets for 22 weeks. N=7-12. **e**) ITT of mice on diets for 20 weeks. N=8-9. **f)** Number of metabolites changed between the indicated diets.

Supplementary Figure S2

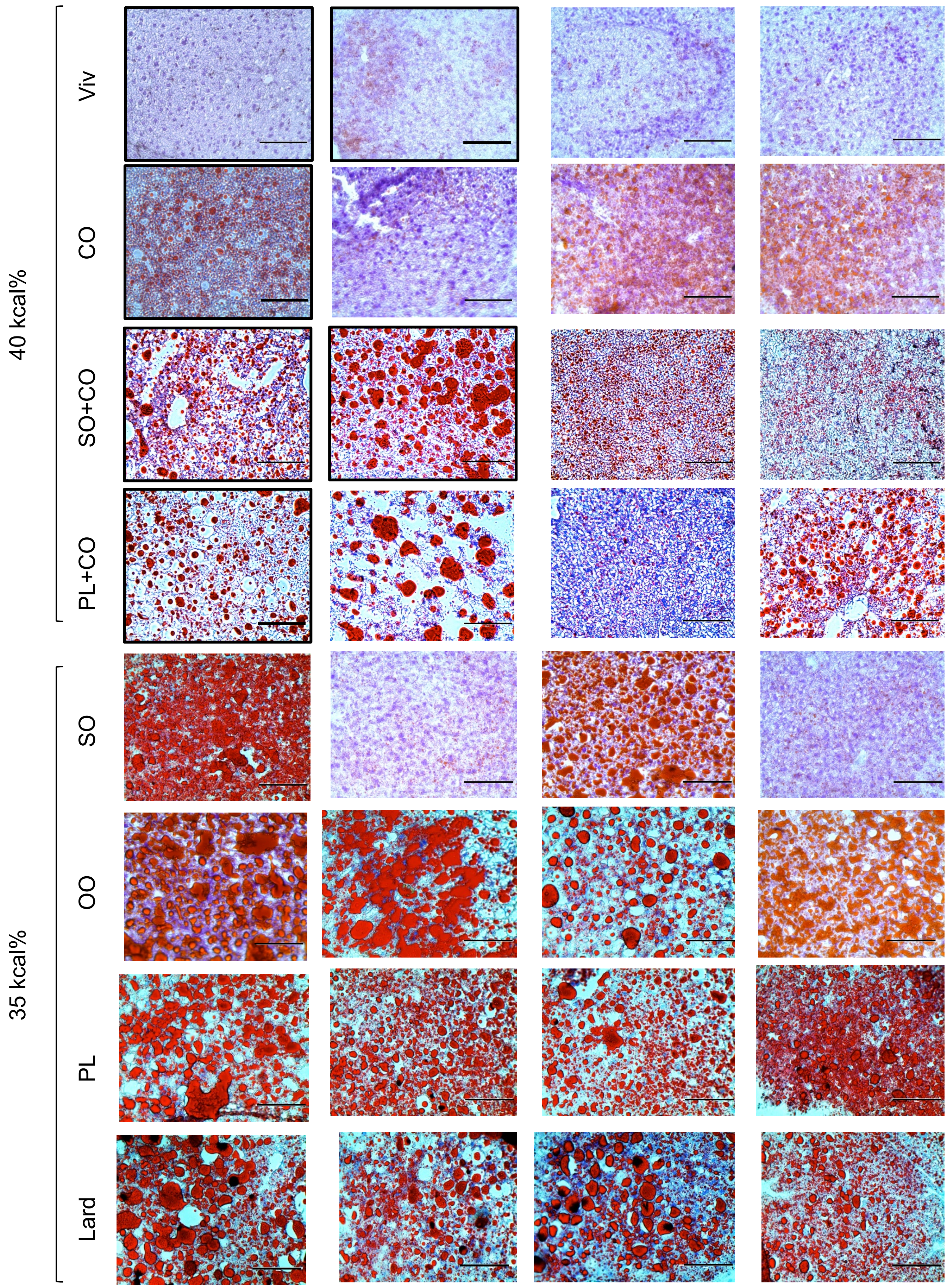

Supplementary Figure S2. Oil Red O staining of liver sections from mice fed the various diets.
Oil Red O staining for fatty liver in C57BL/6N male mice on the various diets for 24 weeks. Scale bars are 100 microns. N= 4-6 mice per diet. Images also shown in Fig. 1f are boxed.

Supplementary Figure S3

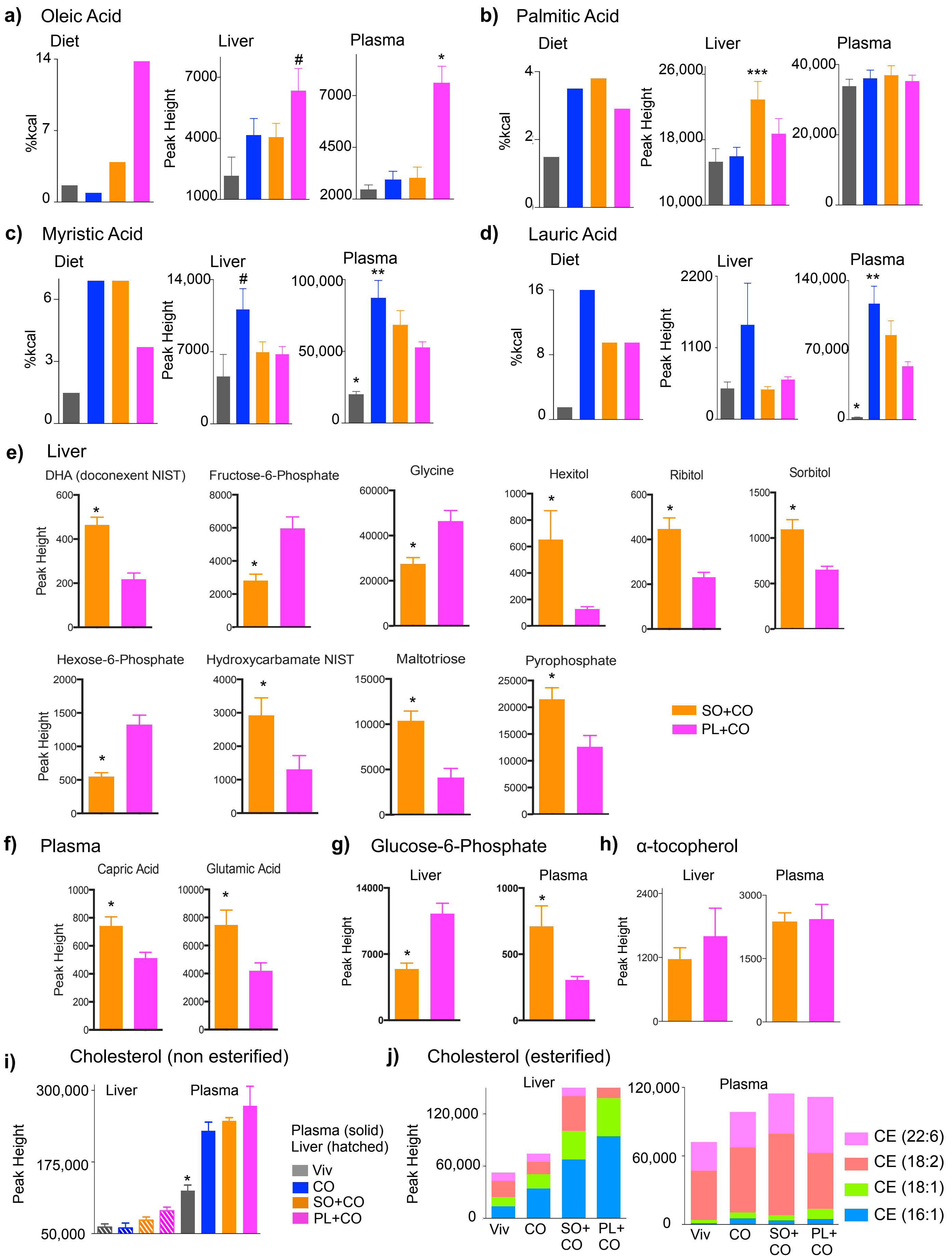

Supplementary Figure S3. Primary metabolites altered by a soybean oil-enriched diet.

a-d) Levels of the indicated fatty acids in the diets and liver and plasma of C57BL/6N male mice fed the respective diets. N=7-8. *Significantly different from all others, **from PL, *** from CO and Viv, # from Viv ($P \leq 0.05$, by One-way ANOVA, Tukey's post-hoc analysis). See Table S1 for values of fatty acids in the diets and Table S2 for values of their metabolites in the liver and plasma. **e-g)** All other significantly different primary metabolites between SO+CO and PL+CO in liver **(e)**, plasma **(f)** and liver and plasma **(g)** where glucose-6-phosphate exhibits opposing trends. **h)** α-tocopherol, a potent anti-oxidant that is typically elevated in conventional soybean oil, showed no alteration in Plenish in either liver or plasma. N=8 mice per group. * Significantly different ($P<0.05$) One-way ANOVA, Tukey's post hoc analysis. **i)** Free cholesterol in the liver and plasma. *Significantly different from others in same tissue ($P \leq 0.05$ by One- way ANOVA, Tukey's post-hoc analysis). **j)** Relative abundance of esterified cholesterol species in liver and plasma. **CE (cholesteryl ester) 16:1**: Viv significantly different from others in liver and plasma; PL+CO different from CO in liver. **CE18:1**: Viv significantly different than all others in liver; PL+CO different from CO in liver; all diets different from each other in plasma. **CE18:2**: SO+CO significantly different from all others in liver and from PL+CO and Viv in plasma. **CE22:6**: SO+CO and PL+CO significantly different from Viv and CO in liver; PL+CO different from Viv in plasma. Significance is $P \leq 0.05$ determined by One- way ANOVA, Tukey's post-hoc analysis.

Supplementary Figure S4

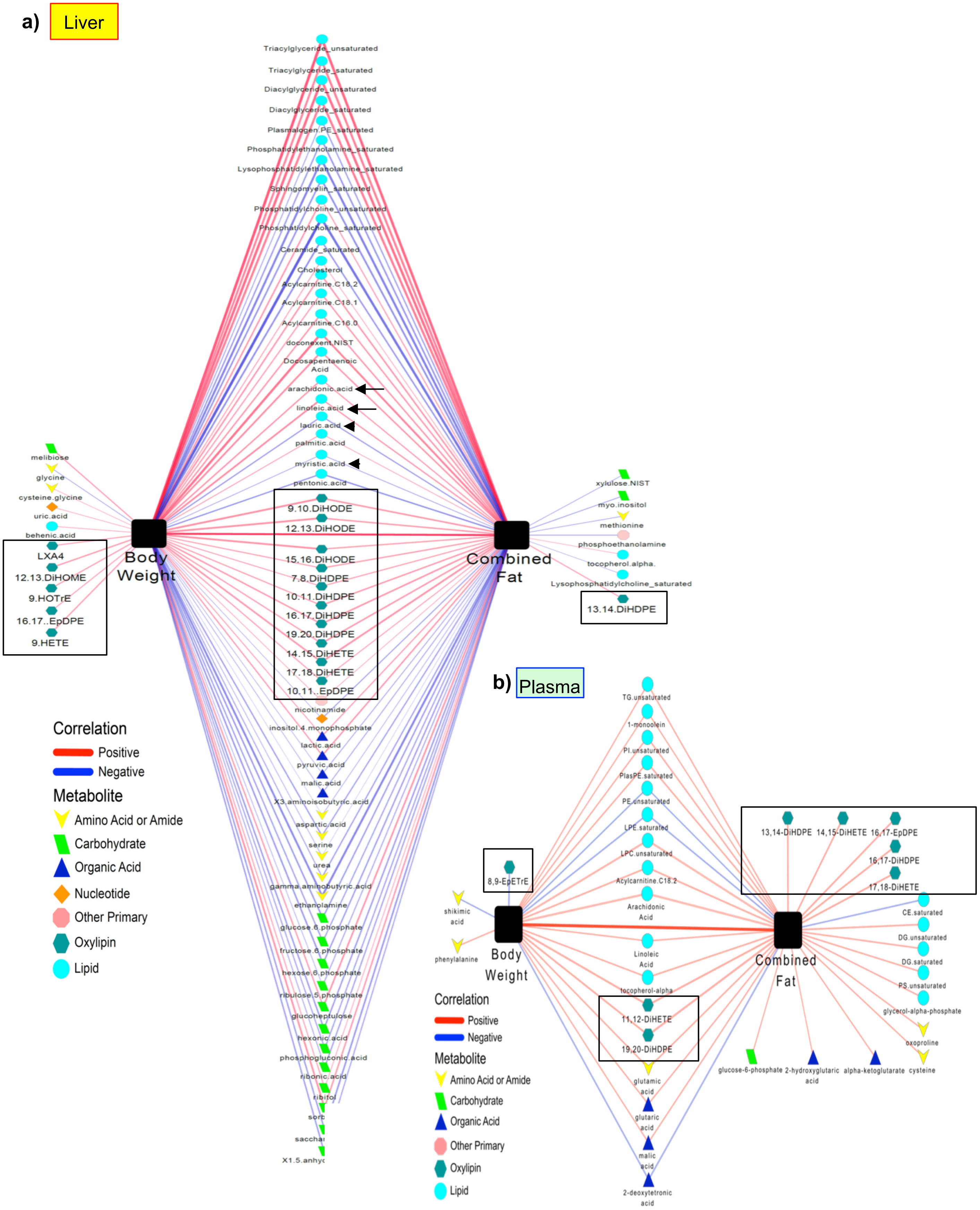

c)

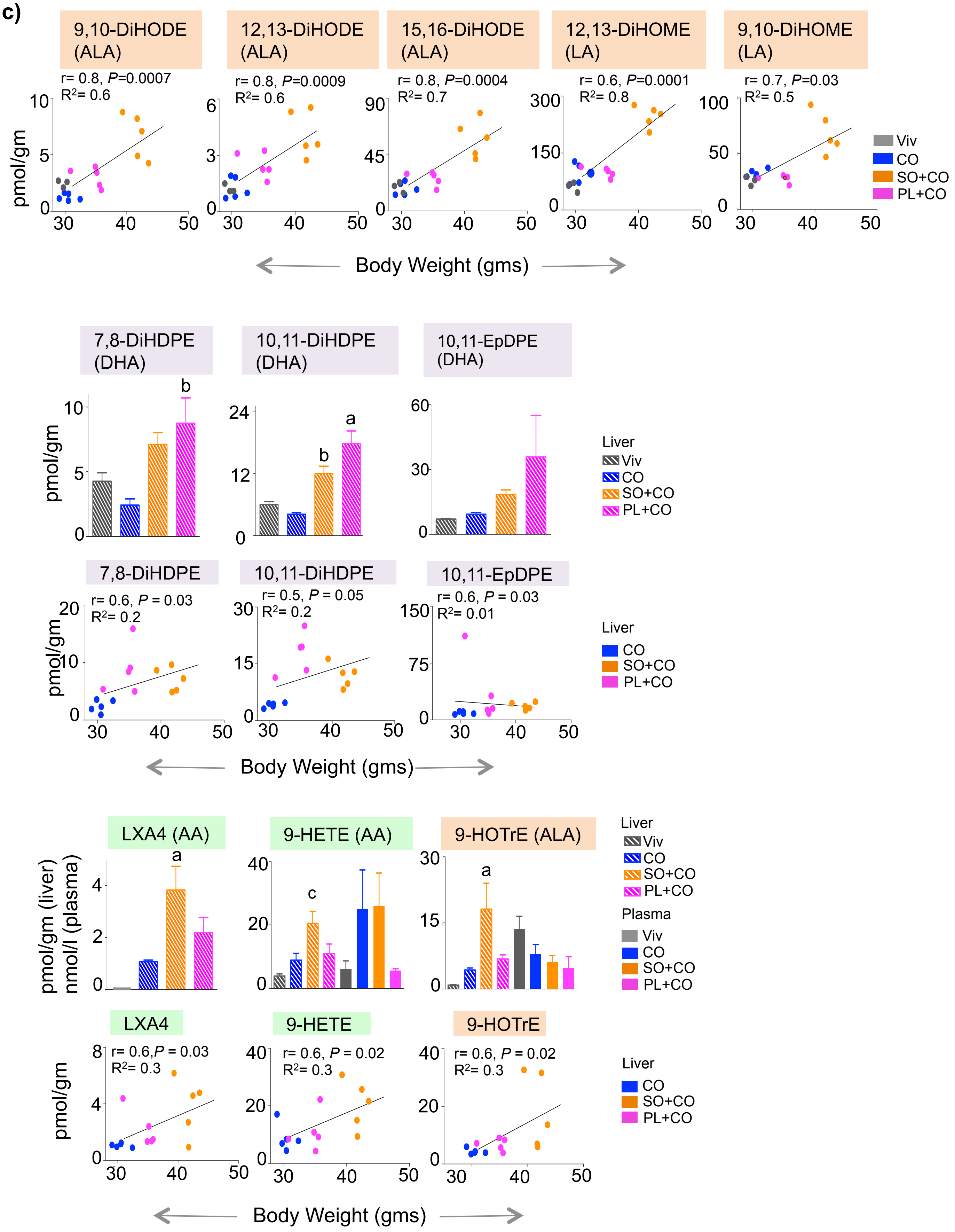

9,10-DiHODE (ALA)
12,13-DiHODE (ALA)
15,16-DiHODE (ALA)
12,13-DiHOME (LA)
9,10-DiHOME (LA)
r= 0.8, P=0.0007 R2= 0.6
r= 0.8, P=0.0009 R2= 0.6
r= 0.8, P=0.0004 R2= 0.7
r= 0.6, P=0.0001 R2= 0.8
r= 0.7, P=0.03 R2= 0.5
pmol/gm
Viv
CO
SO+CO
PL+CO
Body Weight (gms)
7,8-DiHDPE (DHA)
10,11-DiHDPE (DHA)
10,11-EpDPE (DHA)
Liver
7,8-DiHDPE
10,11-DiHDPE
10,11-EpDPE
r= 0.6, P = 0.03 R2= 0.2
r= 0.5, P = 0.05 R2= 0.2
r= 0.6, P = 0.03 R2= 0.01
LXA4 (AA)
9-HETE (AA)
9-HOTrE (ALA)
pmol/gm (liver) nmol/l (plasma)
Plasma
LXA4
9-HETE
9-HOTrE
r= 0.6,P = 0.03 R2= 0.3
r= 0.6, P = 0.02 R2= 0.3
r= 0.6, P = 0.02 R2= 0.3

d) Correlate in both liver and plasma: elevated in SO+CO and PL+CO

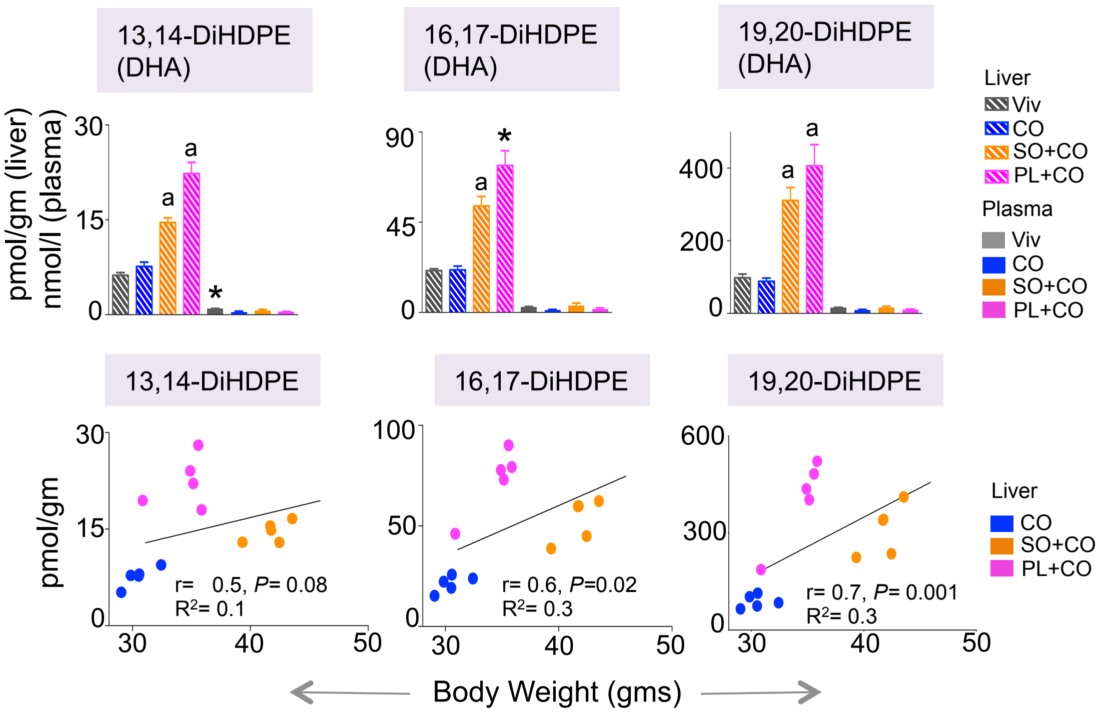

Correlate in both liver and plasma: higher in Viv than SO+CO or PL+CO or no significant difference between diets in the liver

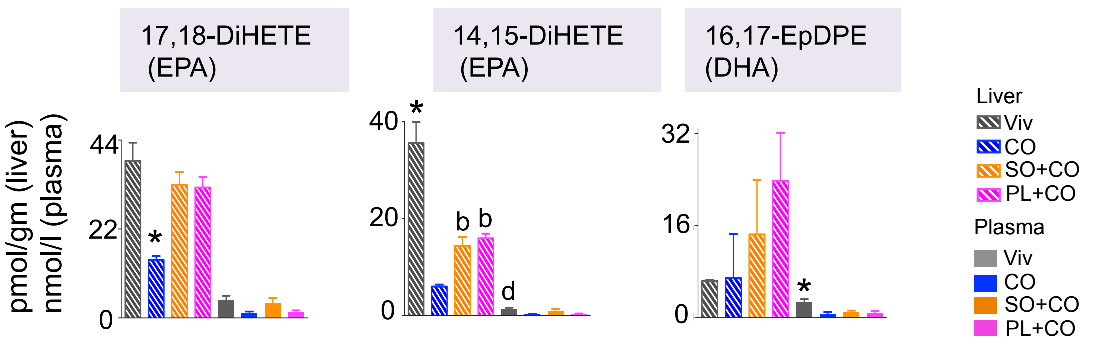

e) Correlate only in plasma

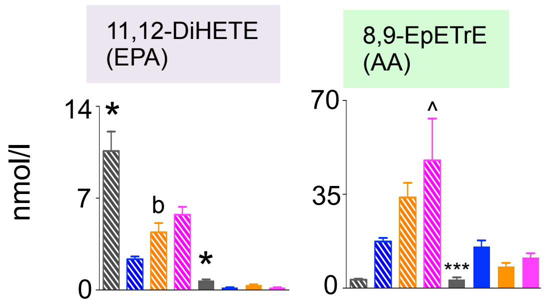

Metabolite	r, *P*-value	R^2
17,18-DiHETE	0.7, 0.004	0.5
14,15-DiHETE	0.6, 0.02	0.4
11,12-DiHETE	0.4, 0.14	0.09
16,17-EpDPE	0.5, 0.04	0.005
8,9-EpETrE	0.4, 0.19	0.001

f) Correlation of fatty acids in the liver with body weight

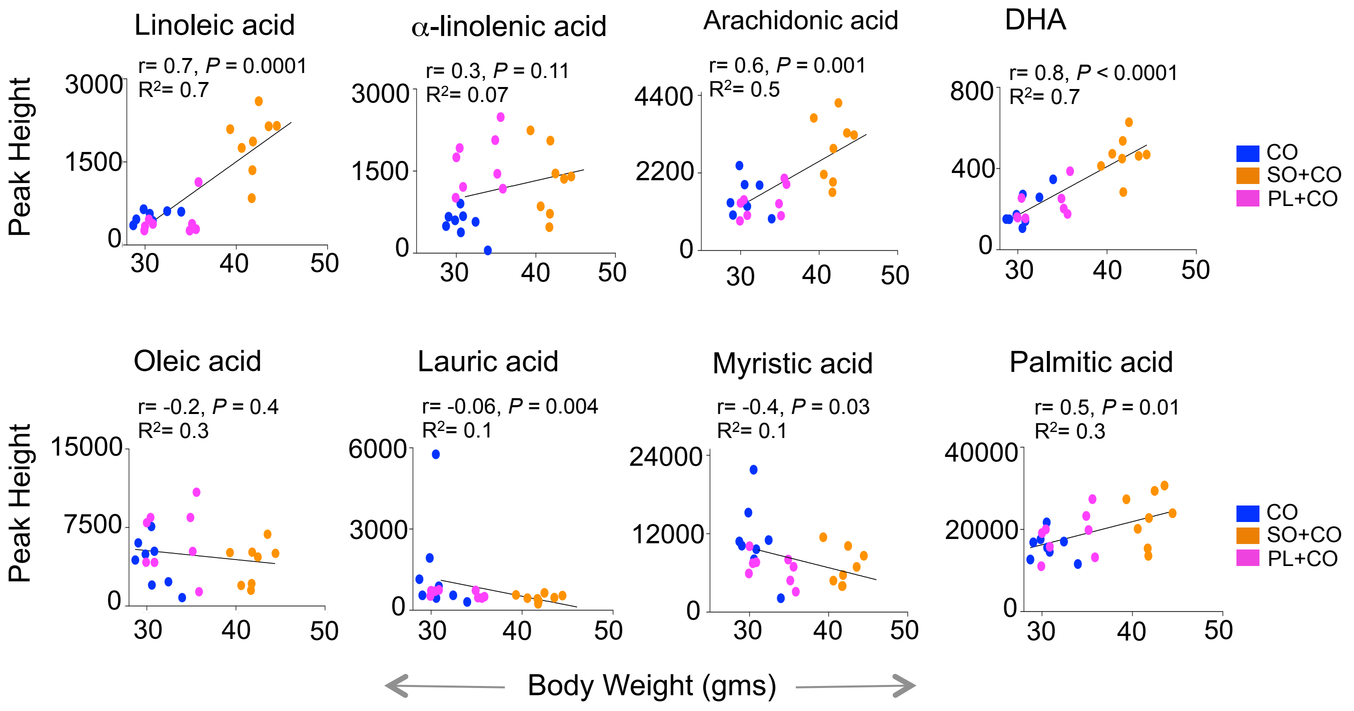

g) Correlation of fatty acids in the liver with body weight – all diets including Viv

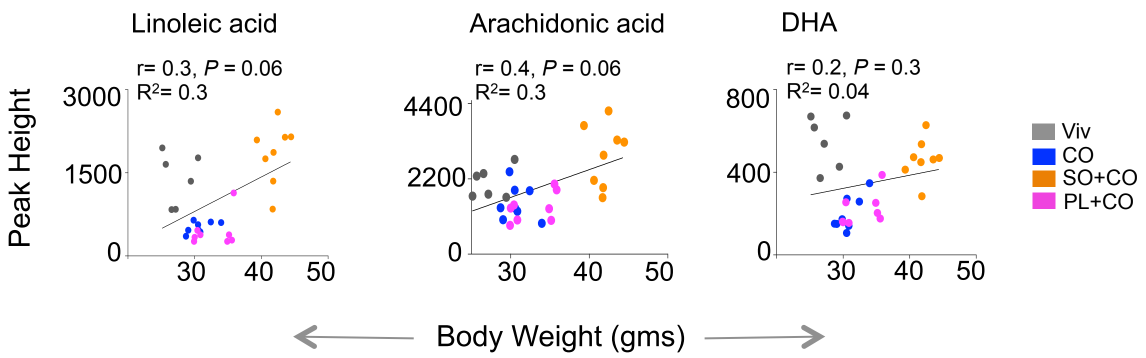

Supplementary Figure S4. Oxylipins correlate with obesity and adiposity in soybean oil-fed mice.
Correlation between phenotype and metabolites of individual C57BL/6N male mice in **a)** Liver and **b)** Plasma of mice fed CO, SO+CO or PL+CO diets. Edge colors depict correlation (red, positive; blue, negative) between metabolites and phenotypic measurements (black squares). Edge width represents strength of the correlation. Node color and shape represent the different classes of metabolites and lipids. Only those samples with full meta-data and significant (P<0.05) Spearman's correlation coefficient were included. Networks were generated using Cytoscape. Box, oxylipins. Metabolites mentioned in the text (arrows, positively correlating; arrowheads, negatively correlating). **c-e)** Correlation graphs between body weight and concentration of oxylipins showing significant correlations in the livers of individual mice. Spearman correlation coefficient (r) and associated P values and R^2 values for linear regression are indicated on the graphs. Levels of oxylipins that correlate with body weight and/or total fat in liver (hatched bars) or plasma (solid bars) or in both tissues as indicated are also included. Legend for (**e**) is same as (**d**). Table in (**e**) shows Spearman correlation coefficient (r) and associated P values and R^2 values for linear regression for indicated oxylipins in the liver. Viv values are included for reference purposes only; it was not included in the correlation or linear regression analyses. In parentheses is the fatty acid from which the oxylipin is generated. N=4-5 mice per group. *Significantly different (within same tissue) from all others, [a] from CO and Viv, [b] from CO, [c] from Viv, [d] from CO and PL+CO, $P\leq$ 0.05. **f, g)** Correlation graphs between body weight and concentration of fatty acids in the liver of individual mice for the high fat diets only (**f**) or with Viv group included (**g**). Spearman correlation coefficient (r) and associated P values and R^2 values for linear regression are indicated on the graphs. N=4-5 mice per group.

Supplementary Figure S5

a)

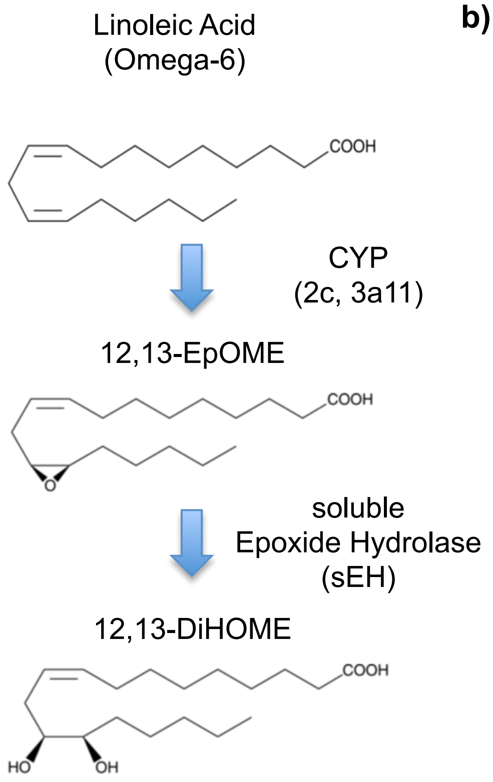

b)

	Plasma (24 wk)		Liver (24 wk)	
Product/ Substrate	SO+CO	PL+CO	SO+CO	PL+CO
12,13-DiHODE/ 12(13)-EpODE	0.41	0.83	0.63	2.04
15,16-DiHODE/ 15,16-EpODE	0.47	0.23	2.26	3.59
9,10-DiHODE/ 9,10-EpODE	0.53	0.30	0.61	1.74
12,13-DiHOME/ 12,13-EpOME	1.11	1.18	1.54	2.74
9,10-DiHOME/ 9,10-EpOME	0.77	0.95	0.48	0.82

c)

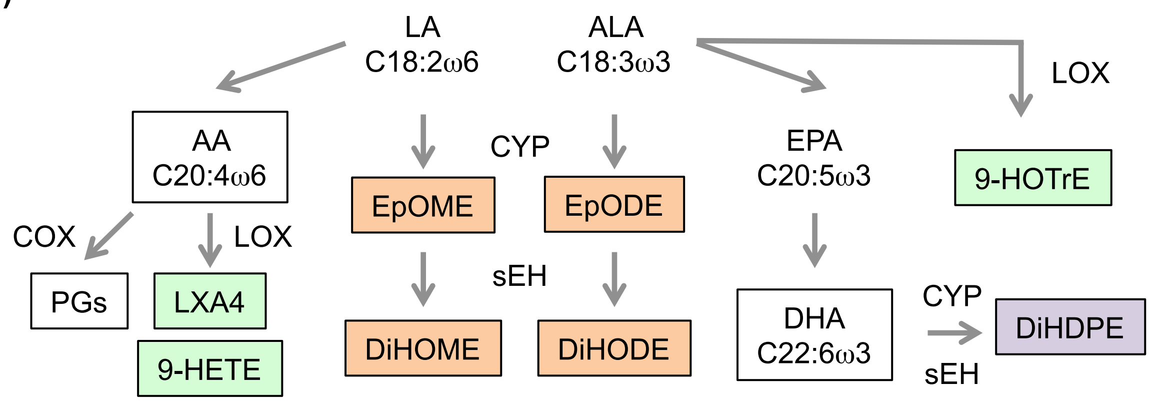

Supplementary Figure S5. Linoleic acid (LA) and α-linolenic acid (ALA) metabolism.
a) Structure of LA and its oxylipin metabolites. **b)** Ratio of oxylipin diol:epoxide as a measure of soluble epoxide hydrolase (sEH) activity. Red, significantly different from corresponding SO+CO ($P \leq 0.05$, *T*-test). N=3-5 per group.
c) LA and ALA metabolites (boxed) featured and color-coded as in Figures 2, 3, 5 and 7.

Supplementary Figure S6

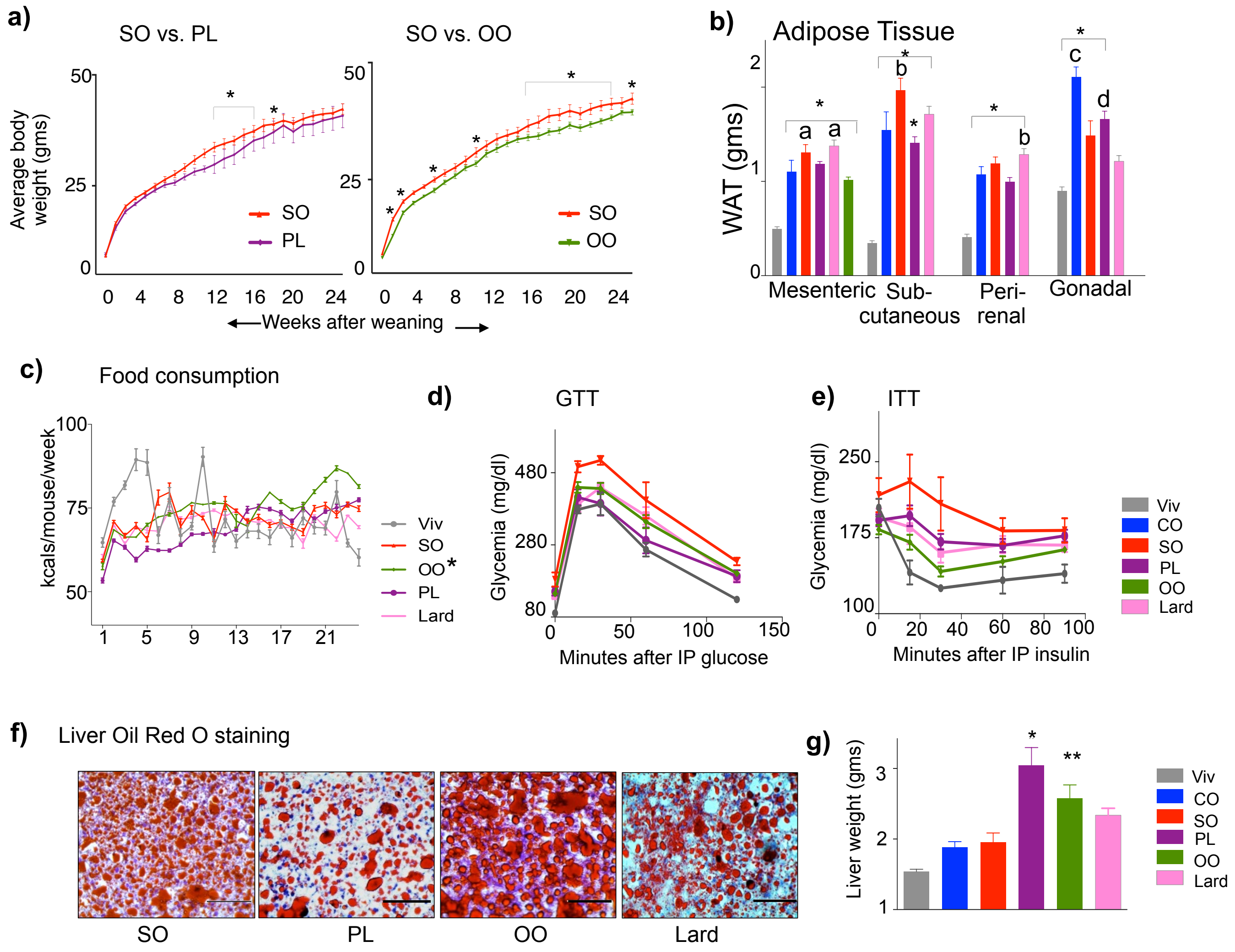

Supplementary Figure S6. Plenish induces similar metabolic effects as olive oil; conventional soybean oil is similar to lard.

a) Average weekly body weights of male C57BL/6N male mice started on the indicated diets at weaning. CO, high fat diet largely from coconut oil; SO, soybean oil only diet; PL, Plenish oil only diet; OO, olive oil only diet. All diets are isocaloric with 35 kcal% total fat except CO which has 40 kcal%. N = 7-16. * SO significantly different from PL or OO. Significance is defined as $P \leq 0.05$ determined by Two-way ANOVA, with Holm-Sidak's multiple comparison test. **b)** Average mass of different types of white adipose tissue from mice on diets for 24 weeks. *All tissues,* * significantly greater than Viv*;* [a] greater than OO*;* [b] greater than PL; [c] CO greater than SO, PL, lard; [d] greater than lard. **c)** Average weekly food consumption of mice on a per cage basis. N = 10-15 mice (3–4 cages) per diet. *Mice on the OO diet consumed significantly more kcals over the course of the experiment (average of 1793 kcals/ mouse, ~75 kcals/week) compared to mice on the PL diet (average of 1649 kcals/mouse, ~69 kcals/week) by One-way ANOVA, Holm-Sidak post hoc analysis (P<0.05). There was no difference in kcals consumed between SO (1718 kcals/mouse; 72 kcals/week) and PL or OO fed mice. **d)** GTT (at 18-20 weeks on the diet). N=4-13. **e)** ITT (at 18 weeks on the diet). N=5-12 except CO (N=3) and Viv (N=4). **f)** Representative Oil Red O staining of livers. Scale bar is 100 microns. N= 4-6 per group. (See **Supplementary Fig. S2** for additional sections). **g)** Liver weight at harvest. * Significantly higher than all others except OO, or ** than SO, CO and Viv. N=10-13.

Supplementary Figure S7. Oxylipins detected in this study and their parent fatty acids.

Oxylipin Species	Parent Fatty Acid
9-HOTrE	ALA
13-HOTrE	ALA
15,16-DiHODE	ALA
9,10-DiHODE	ALA
12,13-DiHODE	ALA
15(16)-EpODE	ALA
9(10)-EpODE	ALA
15-HETE	ARA
11-HETE	ARA
15-oxo-ETE	ARA
8-HETE	ARA
12-HETE	ARA
9-HETE	ARA
5-HETE	ARA
14,15-DiHETrE	ARA
11,12-DiHETrE	ARA
19,20-DiHDPE	DHA
16,17-DiHDPE	DHA
19(20)-EpDPE	DHA
16(17)-EpDPE	DHA
15-HEPE	EPA
8-HEPE	EPA
12-HEPE	EPA
5-HEPE	EPA
17,18-DiHETE	EPA
14,15-DiHETE	EPA
13-HODE	LA
9,12,13-TriHOME	LA
9,10,13-TriHOME	LA
12,13-DiHOME	LA
9,10-DiHOME	LA
9-HODE	LA
9-oxo-ODE	LA
EKODE	LA
12(13)-EpOME	LA
9(10)-EpOME	LA

ALA, alpha-linolenic acid
LA, linoleic acid
ARA, arachidonic acid
DHA, docosahexenoic acid
EPA, eicosapentenoic acid

Supplementary Table S1. Composition of diets and oils used in this study.

	Viv	CO	SO+CO	PL+CO	SO	PL	OO	Lard
**Nutrient**	gm%	gm%	gm%	gm%	gm%	gm%	gm%	gm%
Protein	23.9	20.1	20.1	20.1	19.5	19.5	19.5	19.5
Carbohydrate	48.7	53.4	53.4	53.4	57.5	57.5	57.5	57.5
Fat	5.0	21.5	21.5	21.5	18.2	18.2	18.2	18.2
kcal/gm	*3.36*	*4.87*	*4.87*	*4.87*	*4.26*	*4.26*	*4.26*	*4.26*
**Fat (kcal%)**	**13**	**40**	**40**	**40**	**35**	**35**	**35**	**35**
**Linoleic Acid (kcal%)**	**1.2**	**2**	**10**	**1.4**	**19**	**2.6**	**4.5**	**8**
**Ingredient**		gm	gm	gm	gm	gm	gm	gm
Casein, 80 Mesh		228	228	228	228	228	228	228
DL-Methionine		2	2	2	2	2	2	2
Maltodextrin 10		120	120	120	170	170	170	170
Corn Starch		480	480	480	497.5	497.5	497.5	497.5
Soybean Oil		25	115	0	215	0	0	0
Plenish Oil		0	0	112	0	215	0	0
Coconut Oil, Hydrogenated		220	130	130	0	0	0	0
Olive Oil		0	0	0	0	0	215	0
Lard		0	0	0	0	0	0	215
Mineral Mix S10001		40	40	40	40	40	40	40
Sodium Bicarbonate		10.5	10.5	10.5	10.5	10.5	10.5	10.5
Potassium Citrate, 1 H2O		4	4	4	4	4	4	4
Vitamin Mix V10001		10	10	10	10	10	10	10
Choline Bitartrate		2	2	2	2	2	2	2
**Total kcal%**		**5557**	**5557**	**5557**	**5565**	**5565**	**5565**	**5565**
**Fatty Acid composition of oils**	**Coconut**	**Soybean**	**Plenish**	**Olive**	**Lard**			
Lauric (12:0)	45	<0.05	<0.05	NA	0.1			
Myristic (14:0)	17.5	0.07	<0.05	NA	1.2			
Palmitic (16:0)	8.67	10.6	5.81	11.7	20			
Stearic (18:0)	10.2	3.98	4.17	3.2	11			
Oleic (18:1)	0.25	20.9	73.9	76.5	34.9			
Linoleic (18:2 ω6)	<0.06	52.9	7.42	6	21.9			
α-Linolenic (18:3 ω3)	<0.06	6.54	1.91	0.6	1.3			
**ω6:ω3 (18:2/18:3)**	ND	8.1	3.4	10	16.9			

Supplementary Table S2 is the Metabolomics and Proteomics Dataset that has been uploaded separately as an Excel File titled Supplementary Table S2.

The raw metabolomics data has been deposited on **Metabolomics Workbench** (www.metabolomicsworkbench.org) under Project # PR000461.

Proteomics data for liver tissue has been deposited in the proteomics repository **Massive** http://massive.ucsd.edu with an ID# MSV000081149 and can also be accessed via **Proteome Exchange** with a Proteome Exchange # PXD006681.
